# HO-1 modulates obesity-related renal sodium metabolism via oxidative stress and Na/K-ATPase signaling

**DOI:** 10.1042/CS20257602

**Published:** 2025-11-04

**Authors:** Jiahui Cai, Feifei Sun, Qiaoyun Pan, Shasha Zhao, Yunbo Sun, Feng Yang, Danshu Wang, Runyan Tan, Weiping Liu, Qiang Tan, Xue Shao, Sandrine V. Pierre, Yanling Yan

**Affiliations:** 1 Key Labs Nanobiotech & Applied Chemistry, Department of Biotechnology & Engineering, School of Environmental and Chemical Engineering, Yanshan University, Qinhuangdao, 066004, China; 2 Division of Nephrology, The First Hospital of Qinhuangdao, Qinhuangdao, China; 3 Division of Cardiology, The First Hospital of Qinhuangdao, Qinhuangdao, China; 4 Department of Biomedical Sciences, Joan C. Edwards School of Medicine, Marshall University, Huntington, WV, 25755, U.S.A.; 5 Joan C. Edwards School of Medicine, Marshall University, Huntington, WV, 25755, U.S.A

**Keywords:** HO-1, Na/K-ATPase, obesity, oxidative stress, renal sodium metabolism

## Abstract

The dysregulation of renal sodium metabolism linked to obesity and excessive dietary salt intake is a significant factor in the development of salt-sensitive hypertension. Our previous research has demonstrated that oxidative stress—particularly through the amplification loop of reactive oxygen species (ROS)—plays a critical role in modulating renal sodium handling via Na/K-ATPase signaling. This present study aims to determine whether the antioxidant enzyme heme oxygenase-1 (HO-1) modulates renal sodium metabolism by affecting oxidative stress and the Na/K-ATPase pathway, potentially revealing novel therapeutic avenues. To investigate this, we conducted high-salt dietary interventions and administered Co(III) protoporphyrin IX chloride (CoPP) in both normal and obese C57BL/6J mice. Results indicated that obesity exacerbated oxidative stress and disrupted sodium metabolism. Notably, the induction of HO-1 via CoPP effectively reduced oxidative stress, suppressed inflammatory responses, and modulated mechanisms of renal sodium handling. These observations were corroborated by decreases in protein carbonylation and malondialdehyde (MDA) levels, as well as inhibition of the IL-6/STAT3 inflammatory pathway. Importantly, up-regulation of HO-1 corresponded with a reduction in activated Na/K-ATPase signaling, likely attributable to diminished ROS levels. Furthermore, genetic analyses and urinary metabolite profiles validated the regulatory effects of CoPP on oxidative stress and sodium metabolism. In conclusion, our findings elucidate the dual role of HO-1 as both an antioxidant defense system and a pivotal modulator of sodium excretion. This research underscores the multifaceted physiological functions of HO-1 and its crucial role in regulating renal sodium metabolism, with significant implications for managing salt-sensitive hypertension.

## Introduction

Hypertension is one of the most common chronic diseases, and persistently high blood pressure can damage target organs such as the kidneys and heart, seriously jeopardizing human health [1]. Research has demonstrated a direct link between salt intake and the development of hypertension [2]. In China, the prevalence of hypertension is approximately 27%, with salt-sensitive hypertension accounting for 50% of the reported cases, particularly among the obese population [3,4]. Obese patients with salt-sensitive hypertension exhibit characteristic pathophysiological alterations, particularly dysregulated sodium homeostasis, sympathetic nerve activation, exacerbated oxidative stress, and inflammatory responses [5,6]. Among these, impaired renal sodium handling is recognized as a pivotal mechanism in the progression of salt-sensitive hypertension [7,8].

In studies focusing on renal sodium metabolism, Na/K-ATPase plays a crucial role in maintaining sodium ion concentration in renal cells and regulating overall sodium homeostasis in the body. We have demonstrated that Na/K-ATPase exhibits both pumping and signaling functions [3,6,9]. The signaling ability can amplify reactive oxygen species (ROS) signaling and increase cellular oxidative stress, while ROS generation further activates the Na/K-ATPase signaling pathway through a positive feedback mechanism [10]. The signaling function can regulate sodium transport in the renal proximal tubule (RPT) and inhibit sodium reabsorption in RPT, thereby correcting sodium retention-induced volume expansion and elevated blood pressure. Specifically, ROS can signal through the Na/K-ATPase signaling cascade and modulate renal sodium handling and blood pressure [6].

Notably, dysfunction of the Na/K-ATPase signaling pathway is not only involved in the pathogenesis of hypertension but also provides novel targets for therapeutic intervention. In terms of treatment strategies, multiple studies have focused on endogenous Na/K-ATPase inhibitors. For example, the endogenous digitalis-like compound marinobufagenin is significantly elevated in salt-sensitive hypertensive models, and treatment with anti-marinobufagenin antibodies effectively reduces systolic blood pressure in Dahl salt-sensitive rats [11], suggesting that targeting endogenous inhibitors may represent a feasible therapeutic strategy. On the other hand, studies have also found that hydrogen peroxide (H_2_O_2_), induced by high salt intake, negatively regulates sodium transport in the proximal tubule, thereby partially preventing the development of salt-sensitive hypertension [12]. This highlights the critical role of redox regulation in sodium homeostasis. Current research trends indicate that Na/K-ATPase and its mediated ROS signaling pathway have become central hubs integrating renal sodium metabolism, oxidative stress, and inflammatory responses. Targeting this signaling pathway not only deepens our understanding of the mechanisms underlying hypertension but also holds promise for developing breakthrough therapies for metabolic hypertension and related kidney diseases.

In recent years, heme oxygenase-1 (HO-1) has been identified as a key protective enzyme that plays an important role in regulating renal sodium metabolism and salt-sensitive hypertension, with its mechanisms closely intersecting with the Na/K-ATPase signaling pathway. HO-1 degrades heme to generate carbon monoxide (CO), biliverdin, and free iron, thereby exerting antioxidant, anti-inflammatory, and cytoprotective effects [13]. Studies have shown that HO-1 influences renal sodium handling and ameliorates salt-sensitive hypertension through multiple mechanisms. Specifically, the HO-1/CO system can attenuate high salt-induced hypertension by modulating oxidative stress and pro-inflammatory cytokines (PICs) in the paraventricular nucleus (PVN) [14]. Up-regulation of HO-1 significantly alleviates angiotensin II-induced tubulointerstitial injury and salt-sensitive hypertension [15]. Particularly under salt-loaded conditions, HO-1 up-regulation effectively blocks the Ang II–NADPH oxidase–ROS signaling axis, mitigating oxidative stress-induced dysregulation of sodium transport proteins. Furthermore, induction of HO-1 expression in spontaneously hypertensive rat models with diabetes markedly reduces renal oxidative stress and inflammation levels, further supporting its role in renal protection [16]. In summary, as a critical cytoprotective factor, HO-1 probably plays a significant regulatory role in obesity-related salt-sensitive hypertension by counteracting Na/K-ATPase-mediated dysregulation of renal sodium metabolism through its anti-inflammatory, antioxidant, and signaling modulation functions. Further elucidation of the molecular mechanisms by which HO-1 influences renal sodium handling may provide novel targets and strategies for the prevention and treatment of salt-sensitive hypertension, particularly in the context of obesity.

We hypothesized that HO-1 induction attenuates ROS-mediated oxidative amplification, thereby regulating renal sodium metabolism via the Na/K-ATPase signaling pathway [17]. To test this, obese and normal C57BL/6 J mice were fed a high-salt diet and treated with the HO-1 inducer Co(III) protoporphyrin IX chloride (CoPP) [13]. Normal mice showed appropriate increases in sodium and water excretion in response to high salt, whereas obese mice exhibited impaired sodium handling. CoPP modulated sodium excretion under high-salt conditions. Protein analysis revealed that high salt activated Na/K-ATPase signaling (including phosphorylation of c-Src and ERK1/2), IL-6/STAT3 pathway, and oxidative stress in normal mice. Obese mice displayed heightened oxidative stress and inflammation but no further Na/K-ATPase activation upon salt challenge, suggesting signaling impairment or desensitization. HO-1 up-regulation attenuated oxidative stress, suppressed Na/K-ATPase and IL-6/STAT3 signaling, and supported renal sodium homeostasis, a finding corroborated by cellular experiments.

Moreover, the effects of HO-1 up-regulation on genomic alterations and metabolic reprogramming in obesity-associated renal sodium dysregulation remain incompletely elucidated. In this study, we employed mRNA sequencing and untargeted metabolomics [18] to analyze differentially expressed genes in renal tissues and urinary metabolites from CoPP-treated obese mice. Integrated genetic and metabolomic analyses reveal that HO-1 reprograms oxidative stress responses, sodium transporter activity, and critical metabolic pathways (e.g., porphyrin, glutathione, and amino acid metabolism). These findings establish HO-1 as a central regulator linking oxidative stress, sodium handling, and metabolic dysfunction in obesity. Our work elucidates the regulatory mechanism of HO-1 in renal sodium metabolism and demonstrates its therapeutic potential for obesity-related, salt-sensitive hypertension.

## Materials and methods

### Cell lines

Porcine renal proximal tubule epithelial cells (LLC-PK1) and human renal proximal tubule epithelial cells (HK-2, GNHu47) were obtained from National Collection of Authenticated Cell Cultures. LLC-PK1 medium was formulated with DMEM (Dulbecco’s Modified Eagle’s medium), 10% fetal bovine serum (FBS), 100 units/ml penicillin, and 100 μg/ml streptomycin. HK-2 medium was formulated using Keratinocyte-SFM (Invitrogen, 17005–042) and Gentamicin/Amphotericin Solution (Gibco, R-015–10). Cells were all cultured in a 37°C incubator humidified with 5% CO_2_. The medium was changed regularly until the cell concentration reached 80–90%.

### Animal models

**Figure 1:**
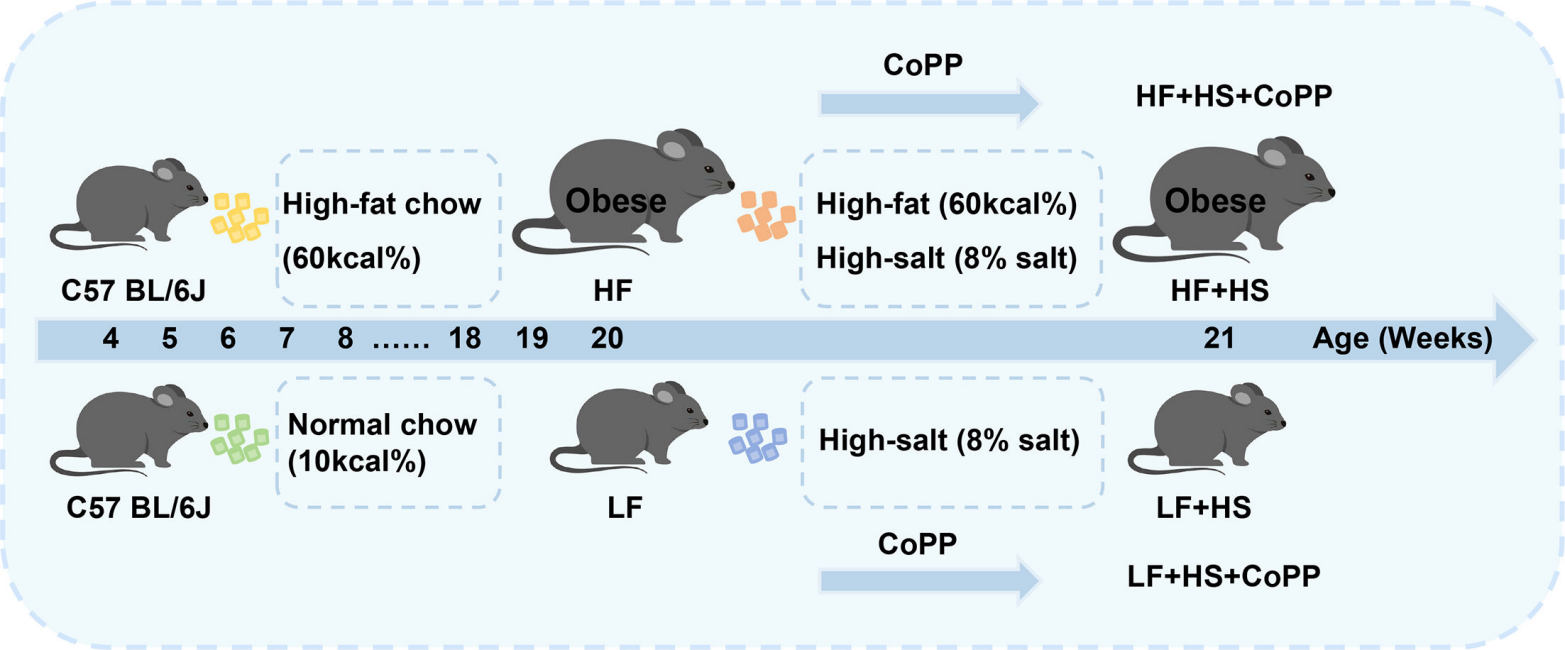
Schematic representation of diet-induced metabolic model in C57 BL/6 J mice.

Four-week-old male C57BL/6 J mice (20 ± 2 g, SPF) were purchased from Beijing Vital River Laboratory Animal Technology Co., Ltd. As shown in Figure 1, all mice were randomly assigned to either a low-fat (LF, 10% kcal) or high-fat (HF, 60% kcal) diet. At 20 weeks, mice were further divided into LF, LF+high salt (HS), LF+HS default CoPP, HF, HF+HS, and HF+HS default CoPP groups. A high-salt diet (8% salt) was provided for 1 week. CoPP (5 mg/kg) was administered intraperitoneally three times weekly. Male C57BL/6 J mice were socially housed in groups under a controlled environment (temperature: 21–23℃; humidity: 40–55%) with a 12 h light/dark cycle and ad libitum access to food and water. All mouse diets were purchased from Shanghai Yitong Biotechnology Co., Ltd.

For terminal procedures, mice were anesthetized with urethane (1.5 g/kg, intraperitoneally) and placed in a supine position on a custom surgical platform equipped with a heating pad to maintain body temperature at 37 ℃. Adequate depth of anesthesia was confirmed by monitoring respiration rate, heart rate, and the absence of a hind-paw withdrawal reflex. Urethane was selected due to its minimal impact on cardiovascular and renal functions during prolonged surgical procedures. At the end of the experiment, mice were killed without recovery from anesthesia. The killing of mice was performed by exposure to an overdose of carbon dioxide (CO_2_) followed by exsanguination and diaphragm transection to ensure death, in accordance with institutional and national ethical guidelines.

The body weight of the mice was recorded weekly, and body length was measured during the final week. Following euthanasia, the kidneys and adipose tissue were promptly dissected and excised. Surface blood and residual fluids were removed, and tissue weights were recorded. The length of the right tibia (TL, mm) was measured. Lee’s index and the abdominal fat index were calculated using the following formulas:


(1) $$Lee^{\prime }s\;index=\frac{\sqrt[{3}]{m_{b}\times 1000}}{L}$$

(2) $$Abdominal\;fat\;index=\frac{m_{f}}{m_{b}}$$

$m_{b}$ is the mouse body weight (g)， L is the mouse body length (cm)， $m_{f}$ is the mouse’s fat weight (mg).

Fasting blood glucose (FBG) was measured via tail clipping [19]. Post high-salt or CoPP treatment, 24 h urine was collected using metabolic cages. Mice were anesthetized, and blood was drawn via cardiac puncture. Kidney cortex/medulla proteins were extracted for Western blot analysis. Kidney tissues were fixed in 4% paraformaldehyde for pathology and immunohistochemical (IHC) staining.

This study used male C57 BL/6 J mice to minimize hormonal variability and ensure model sensitivity, consistent with prior research [3,17]. While this approach enhances experimental reproducibility and historical comparability, we acknowledge that the exclusion of female mice represents a limitation to the generalizability of our findings. Future studies should validate these results in female models to assess potential sex-dependent differences in HO-1-mediated renal protection.

### Renal Na^+^ handling studies

The flame photometric method (PerkinElmer, PinAAcle 900T) was used to determine the concentrations of Na^+^ and K^+^ in plasma and urine samples. The levels of blood urea nitrogen (BUN), creatinine (CRE), superoxide dismutase (SOD), malondialdehyde (MDA) in plasma or urine were assessed using assay kits (BUN, C013-2-1; CRE, C011-2-1; SOD, A001-1-2; MDA, A003-1-2, Nanjing Jiancheng Bioengineering Institute). Creatinine clearance (Ccr), urinary Na^+^ excretion rate (UNa^+^V), fractional Na^+^ excretion rate (FENa^+^), and fractional H_2_O excretion rate (FEH_2_O) were calculated from the following formula [3]:


(3) $$Ccr\left(ml/min\right)=\frac{Ucr\times 24h\;urine\;volume}{Pcr\times 24\times 60}$$

(4) $$UNa^{+}V(mmol/24h)=UNa^{+}\times 24hurinevolume$$

(5) $$FENa^{+}\text{(}\%)=\frac{UNa^{+}\times Pcr}{PNa^{+}\times Ucr}\times 100$$

(6) $$FE_{H_{2}O}(\%)=\frac{24hurinevolume}{Ccr\times 24\times 60}\times 100$$

Ucr and Pcr are the creatinine concentrations in urine and plasma, respectively. UNa^+^ and PNa^+^ represent the concentrations of Na^+^ in urine and plasma, respectively.

### Western blotting

LLC PK1 Cells were treated with CTS (Ouabain, 0.1 μM or Digoxin, 0.1 μM) to determine Src, ERK1/2, and STAT3 phosphorylation and protein carbonylation; some cells were pre-treated with CoPP (2 μM, 24 h). The renal cortex tissue lysates were prepared to determine ACE2 and HO-1 expression, Src, ERK1/2, and STAT3 phosphorylation, and protein carbonylation. The above samples were analyzed by Western blotting using the same procedure as we described [20]. The signal density was analyzed using Image J 1.53 t software. The antibodies used included HO-1 (Enzo, ADI-SPA-895-F), ACE2 (RD systems, AF933), p-Src (Cell signaling, 4943S), Src (Cell signaling, 2108S), p-STAT3 (Cell signaling, 9145S), STAT3 (Cell signaling, 9139S), p-ERK1/2 (Cell signaling, 4370S), ERK1/2 (Cell signaling, 9102S), and β-Actin (SCBT, sc-47778). 2,4-dinitrophenylhydrazine (DNPH) and antibodies against 2,4-dinitrophenyl (DNP) hydrazone derivatives were from Sigma-Aldrich.

### Histopathological staining

The harvested kidney tissues were fixed in 4% paraformaldehyde for 48 h, followed by paraffin embedding and sectioning. Paraffin-embedded kidney sections were subjected to H&E, Masson’s trichrome, Sirius Red, and IHC staining. The kidney tissue sections were digitally scanned using a whole-slide scanner (Pannoramic MIDI, 3DHISTECH). Tissue images were magnified and captured using CaseViewer software at both 40× and 63× magnification (with 63× being the maximum available magnification). Quantification of positively stained areas was performed using Image J software (version 1.53 t).

### Intracellular reactive oxygen species detection

The cellular production of ROS was assessed using the DCFH-DA fluorescent probe. The DCFH-DA assay kit was obtained from Servicebio (Wuhan, China). Cells in the logarithmic growth phase were treated with pharmacological agents (Ouabain or Digoxin, 0.1 μM, 1 h; pre-treated with CoPP, 2 μM, 24 h). Following the manufacturer’s protocol, cells were incubated with the DCFH-DA probe. Representative fluorescent images were acquired using a confocal laser scanning microscope (DP73, OLYMPUS, Japan). Quantitative analysis of the ROS-associated green fluorescence was performed using both a multimode microplate reader (Varioskan LUX, Thermo Fisher Scientific, U.S.A.) for bulk measurement and a flow cytometer (Legend-tek, EVA0206, China) for single-cell analysis.

### Single-nuclei isolation and library preparation

Nuclei isolation from kidney samples was performed according to the ‘Frankenstein’ protocol. The tissue was minced into rice-grain-sized fragments, and cold Nuclei EZ Lysis Buffer was added for homogenization. The homogenate was filtered, and nuclei were pelleted by centrifugation, followed by resuspension in fresh lysis buffer for a second round of incubation and centrifugation. The nuclei were washed with wash buffer, resuspended in DAPI buffer, and filtered through a cell strainer. The nuclear suspension was loaded onto a 10X Genomics Chromium microfluidic chip, and single-cell barcoding was performed using the Chromium Controller system. RNA was reverse-transcribed, and sequencing libraries were constructed using the Chromium Single Cell 3’ v3 kit. Sequencing was carried out on the Illumina NovaSeq 6000 platform.

### Bioinformatics analysis

Following alignment to the mouse mm10 reference genome and initial preprocessing using Cell Ranger, the single-nucleus RNA sequencing data underwent rigorous quality control and downstream analysis using Seurat. Low-quality nuclei were filtered out based on the following criteria: retention of nuclei with at least 200 detected genes and exclusion of those with mitochondrial gene content exceeding 5%. Potential doublets were identified and removed using DoubletFinder. The filtered expression data were log-normalized, and the top 2000 highly variable genes were selected for dimensionality reduction through principal component analysis (PCA). Graph-based clustering and UMAP visualization were performed using the most informative principal components. Cell types were annotated through manual assessment of well-established canonical marker genes.

To identify transcriptomic alterations across conditions, differential expression analysis was carried out using the FindMarkers function in Seurat, with significance thresholds set at an adjusted *P* value < 0.05 and an absolute log2 fold change greater than 0.5. The results were visualized using volcano plots and dot plots to highlight significantly dysregulated genes. Furthermore, functional interpretation of the differentially expressed genes was conducted through KEGG pathway enrichment analysis using the clusterProfiler package in R.

### Metabolomics analysis

Urine samples from HF + HS and HF + HS default CoPP groups (*n* = 6) were thawed at 4℃. Then, 100 μl of urine was mixed with 400 μl of 80% methanol, vortexed, and then placed on ice for 5 min. The mixture was then centrifuged at 15,000 g (4℃, 20 min). Supernatant was diluted to 53% methanol with MS-grade water, centrifuged again, and analyzed by LC-MS [21]. Novogene used a Q Exactive™ HF/Q Exactive™ HF-X mass spectrometer and Vanquish UHPLC chromatography (Thermo Fisher, Germany) for analysis.

Data processing was conducted on Linux (CentOS 6.6) using R and Python. Metabolites were annotated by matching with the Human Metabolome Database (HMDB, https://hmdb.ca/metabolites), the LIPIDMaps database (http://www.lipidmaps.org/), the KEGG database (https://www.genome.jp/kegg/), and the MassList database (a self-constructed database of Beijing Novogene Co., Ltd.). For the multivariate statistical analysis part, the metabolomics data processing software metaX [22] was used to transform the data and then perform principal component analysis (PCA) and unsupervised partial least squares discriminant analysis (PLS-DA) to identify differential metabolites associated with subgroups. And a permutation test was used to ensure the validity of the model. Differentially altered metabolites were screened for positive and negative ion patterns based on univariate analysis. Differential metabolite screening was based on criteria of VIP >1.0, FC >1.2, or FC <0.833, and *P* value < 0.05, as visualized via volcano plots. The KEGG database was utilized to investigate metabolite function and metabolic pathways, with differential abundance scores illustrating average and overall metabolite changes in the enrichment pathways.

### Statistical analysis

Statistical analyses were performed using GraphPad Prism version 9.0. For comparisons between two groups, two-tailed unpaired Student’s *t*-tests were applied. Data involving multiple groups were analyzed using either one-way ANOVA or two-way ANOVA, as appropriate. Two-way ANOVA was employed to assess the main effects of obesity status and treatment, as well as their interaction, followed by Tukey’s post hoc test for multiple comparisons. Where only one independent variable was analyzed, one-way ANOVA with Tukey’s post hoc test was used. Data are presented as mean ± standard deviation (SD) or mean ± standard error of the mean (SEM), as indicated in the figure legends. Significance levels are denoted as follows: *P<0.05, **P<0.01, ***P<0.001, ****P<0.0001; ns, not significant.

## Results

### Characteristics of obese C57 BL/6J mice

**Figure 2:**
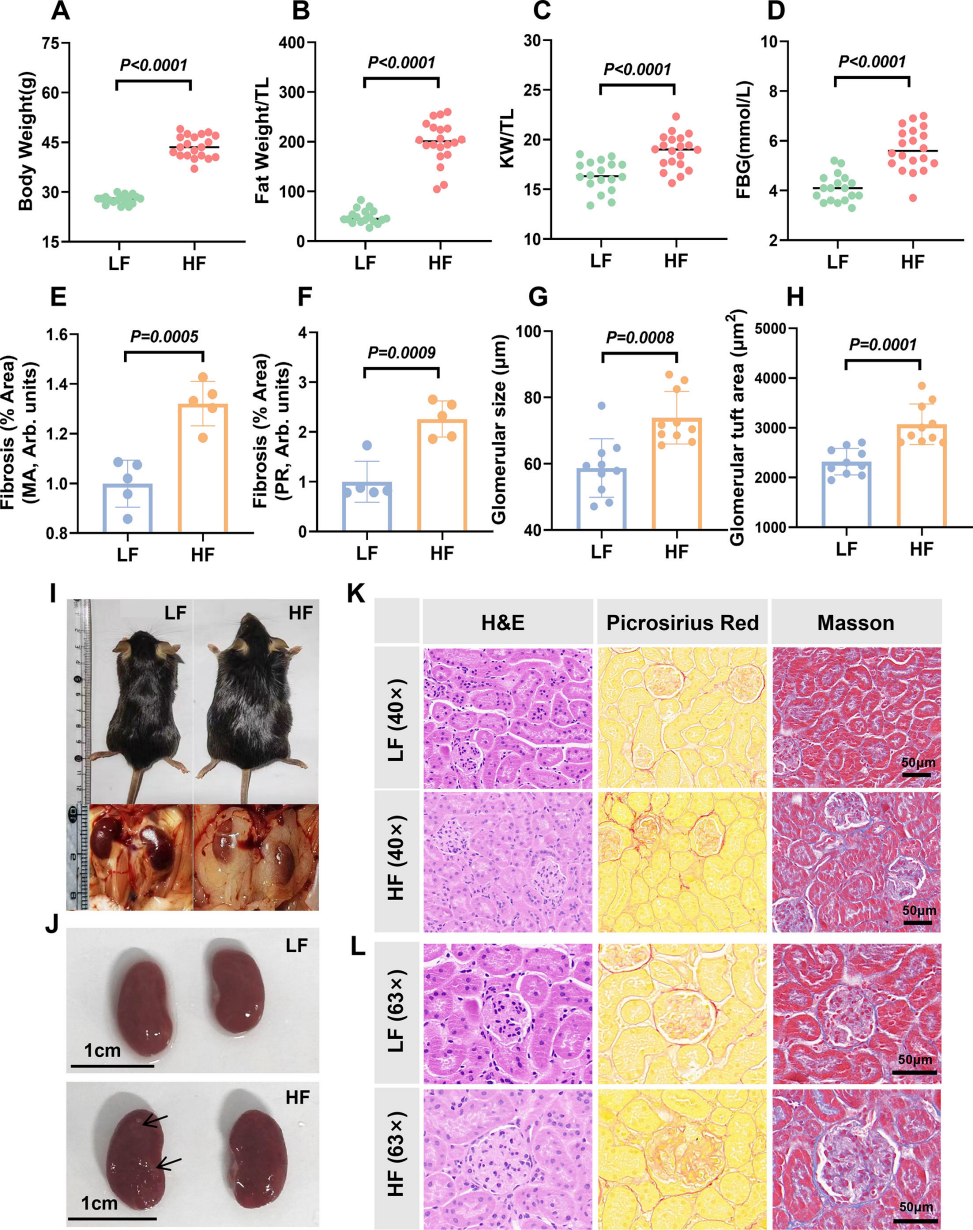
Characteristics of an obese C57 BL/6 J mouse model. **A-D**. Body weight (BW), fat weight/TL (FW/TL), kidney weight/TL (KW/TL) and fasting blood glucose (FBG) were compared between control and obese mice; **E,F**. Representative and quantitative assessment of the areas of extracellular matrix in the glomerulus and tubulointerstitial fibrosis; **G,H**. Quantitative measurement of glomeruli by their external diameter and glomerular tuft area; **I,J**. Representative photographs of the abdomen and kidneys of mice (arrows show fibrosis); **K**. Representative kidney staining images (H&E, Picrosirius Red, Masson) from both mouse groups. **L**. Representative glomerular images from both mouse groups. The statistical significance was calculated using the unpaired, two-tailed, Student’s *t*-test. The data were expressed as the means ± SD, LF (*n* = 18), HF (*n* = 20). *n* = 5 in **E,F**. *n* = 10 in **G,H**.^***^ *P*<0.001,^****^ *P*<0.0001. The tissue images in K,L were magnified and captured using CaseViewer software at both 40 × and 63 × magnification, as shown in Materials and Methods section.

After 16 weeks of being fed on a high-fat chow, the average body weight of the obese mice was significantly higher (by 57.6%) than that of control mice (Figure 2A ). Mice in the obese group also exhibited significantly greater fat weight/tibia length ratio (FW/TL) (Figure 2B), with their total fat weighing approximately four times that of the control mice. Notably, there were extensive fat deposits around the kidneys, referred to as adipose kidneys (Figure 2), and the kidney weights of obese mice were significantly larger than those of the control group, showing a 15.9% increase in kidney size (kidney weight/TL) (Figure 2C J).

Additionally, fasting blood glucose levels were significantly higher in obese mice compared with the control group (Figure 2D). Histological staining of the kidneys revealed that the kidneys of obese mice exhibited partial fibrosis and glomerular hypertrophy (Figure 2H, K L). These findings imply that factors linked to obesity-associated metabolic syndrome could promote hyperglycemia and induce renal pathological changes.

### Obesity and CoPP affect renal sodium metabolism

To investigate the effects of obesity and CoPP on renal sodium metabolism, control and obese mice were randomly assigned to groups for high-salt diet feeding and CoPP treatment (Figure 1). The basic metrics for the mice in each group are presented in Supplementary Table S1. After one week of a high-salt diet, a slight increase in body weight was observed in the mice. CoPP treatment resulted in a reduction in fasting blood glucose levels, although the difference did not reach statistical significance (Supplementary Figure S2).

**Figure 3:**
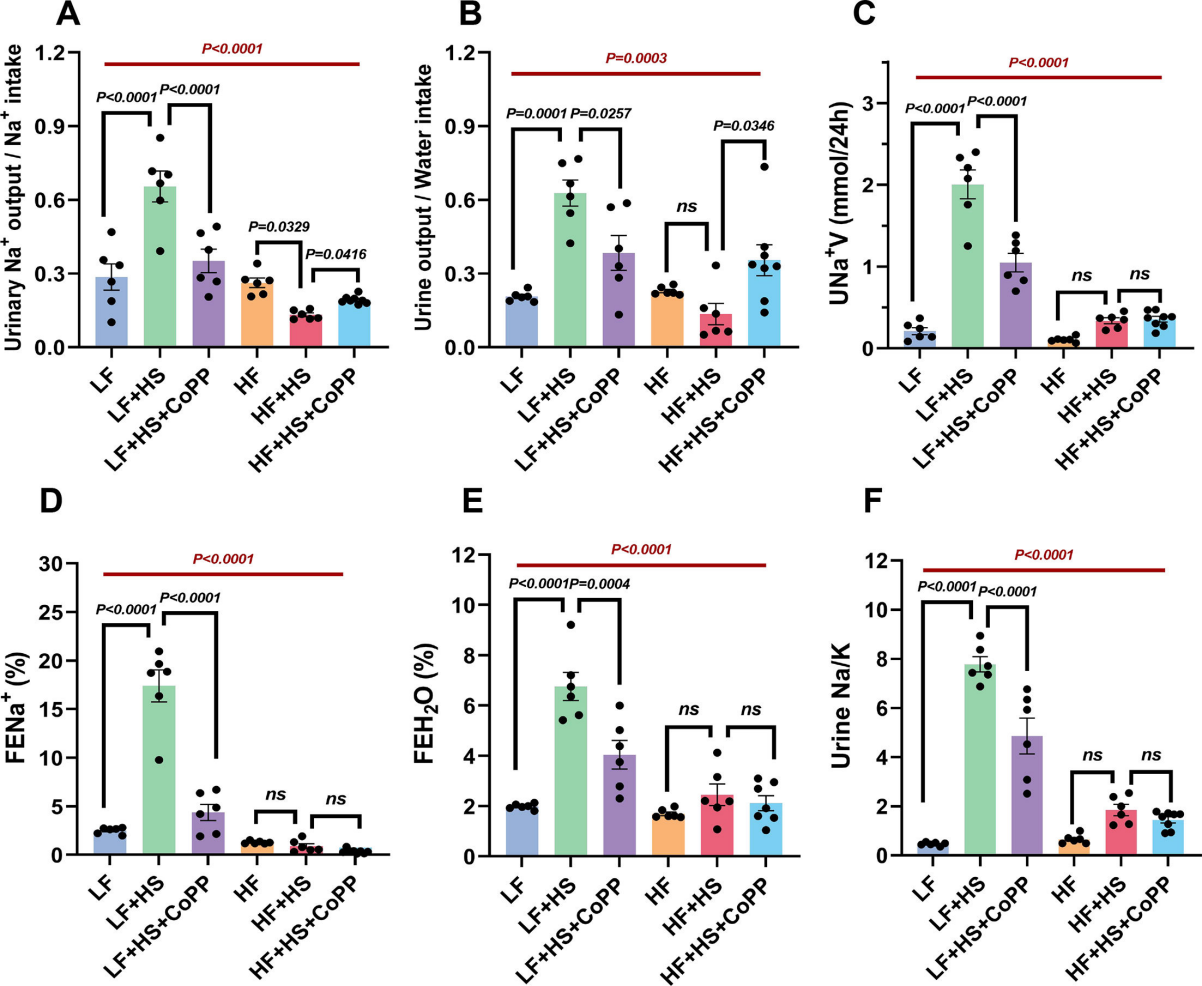
Effects of CoPP treatment on renal sodium metabolism in normal and obese C57BL/6 J mice fed a high-salt diet. (**A**) Urinary Na^+^ output/Na^+^ intake ratio, (**B**) urine output/water intake ratio, (**C**) urinary Na^+^ excretion rate (UNa^+^V), (**D**) fractional Na^+^ excretion (FENa^+^), and (**E**) fractional water excretion (FEH_2_O) in control and obese mice after 1 week high-salt diet and CoPP intervention. (**F**) The concentration ratio of Na^+^/K^+^ (Na^+^/K^+^) in the urine. Data represent mean  ±  SEM. The two-way ANOVA with Tukey’s post hoc comparison was used for statistical significance. The red highlighting represents the overall difference between the control and obese mouse groups. *n* = 6 default 8. ^****^ *P*<0.001, ^***^ *P*<0.001, ^**^ *P*<0.01, ^*^ *P*<0.05, respectively.

Analysis of plasma and urinary parameters revealed that normal mice on a high-salt diet (LF+HS) exhibited significantly increased sodium and water excretion compared with the control group (LF), as evidenced by elevated urinary Na^+^ excretion, urine output, FENa^+^, and FEH_2_O (Figure 3A–3E). This response reflects the body’s autoregulatory mechanism to handle excessive salt intake. However, obese mice failed to exhibit significant increases in sodium and water excretion following high-salt loading (Figure 3A–3E **, S3**), indicating impaired renal sodium handling capacity. This dysregulation suggests a defect in sodium metabolism in obesity. Additionally, the ratio of urinary excreted Na^+^ to K^+^ concentration (Na^+^/K^+^) in the LF+HS group was significantly elevated. In contrast, the Na^+^/K^+^ ratio in the urine of the HF + HS group showed only a slight increase (Figure 3F). Measuring 24 h urinary sodium and potassium is crucial for assessing renal tubular function [23,24]. The observed down-regulation of Na^+^/K^+^ supports the presence of impaired renal sodium metabolism in obese mice.

A high-salt diet causes excessive sodium consumption, leading to increased blood volume. To maintain normal blood volume and osmolality, the kidneys reduce water reabsorption by regulating the water-sodium balance, resulting in increased urine volume [25]. Consequently, the dilution of CRE and urea nitrogen concentrations in the urine of mice in the LF + HS group was observed. Due to the increased urine output in the LF + HS group of mice, the excretion of urea nitrogen over 24 hours was significantly elevated (Supplementary Table S2). Furthermore, plasma urea nitrogen content was significantly lower in the HF + HS mice compared with the LF + HS group. This decrease was attributed to an elevated glomerular filtration rate (GFR) in pathological conditions, which enhances the filtration of metabolic wastes, such as urea nitrogen, leading to a relative decrease in urea nitrogen in plasma [26]. Creatinine clearance (Ccr, used as an index of GFR) was significantly higher in obese mice compared with control mice, indicating that the ability to maintain Na^+^ metabolic homeostasis might be dependent on elevated GFR.

Compared with high-salt-fed normal mice (LF + HS), CoPP treatment significantly attenuated sodium and water excretion (Figure 3A–3E). However, in obese mice, CoPP restored renal sodium excretion capacity, as evidenced by increased urinary Na+excretion and urine output (Figure 3A and B). This bidirectional regulation of renal sodium metabolism by HO-1 is likely mediated through modulation of the Na/K-ATPase signaling pathway. To further elucidate the molecular mechanisms underlying CoPP-mediated regulation of renal sodium metabolism, we systematically evaluated oxidative stress markers, inflammatory mediators, and Na/K-ATPase signaling function in both plasma and renal tissues.

### Obesity disturbs renal sodium metabolic pathways

**Figure 4:**
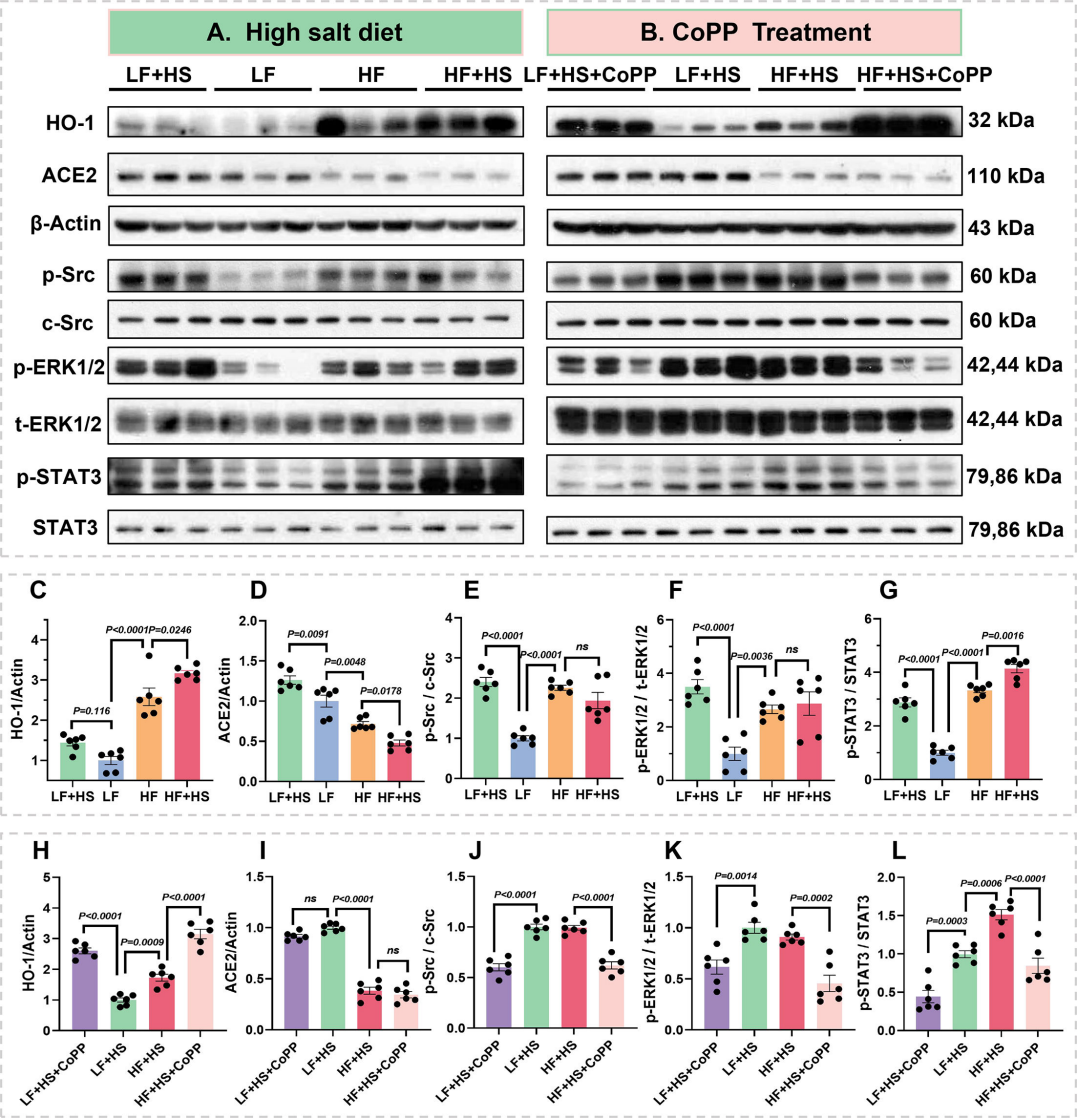
High salt diet and CoPP regulate Na/K ATPase-associated signaling pathways involved in renal sodium homeostasis. **A-B**. Representative Western blots of HO-1, ACE2, phospho-c-Src (p-Src), phospho-ERK1/2 (p-ERK1/2), and phospho-STAT3 (p-STAT3) in renal cortical homogenates. **C-L**. Quantitative analysis of HO-1 expression (**C, H**), ACE2 expression (**D, I**), p-Src / total Src ratio (**E, J**), p-ERK1/2 / total ERK1/2 ratio (**F, K**), and p-STAT3 / total STAT3 ratio (**G, L**). Data represent mean  ±  SEM; the two-way ANOVA with Tukey’s post hoc comparison was used for statistical significance. *n* = 6. ^****^ *P*<0.001, ^***^ *P*<0.001, ^**^ *P*<0.01, ^*^ *P*<0.05, respectively.

Western blots detected activation of sodium metabolic pathways in renal cortical tissues. Our findings revealed that the expression of HO-1 was significantly up-regulated in the kidneys of obese mice compared with those of control mice (Figure 4A and C). This change may represent an adaptive response to obesity-induced renal injury, helping to alleviate oxidative stress and inflammation and exerting a renoprotective effect. Angiotensin-converting enzyme 2 (ACE2), a negative feedback regulator of the renin-angiotensin system, was significantly down-regulated (Figure 4A and D). This down-regulation could lead to a relative increase in Ang II, causing pathological changes such as renal vasoconstriction and intraglomerular hypertension [27]. Furthermore, a high-salt diet significantly activated the Na/K-ATPase signaling pathway in normal mice, as evidenced by increased phosphorylation of Src and ERK1/2 (Figure 4A, E and F), indicating its crucial role in regulating sodium metabolism during high-salt intake. Obese mice also exhibited elevated basal phosphorylation of Src and ERK1/2 in renal tissues, suggesting constitutive activation of Na/K-ATPase signaling. However, high-salt feeding failed to further activate this pathway in obese mice, likely due to signal transduction impairment or desensitization.

Representative IHC images demonstrated similar results. In normal kidneys, HO-1 was weakly expressed in renal tubular epithelial cells, indicating basal levels under normal physiological conditions. However, in obese mice, HO-1 expression expanded to the entire cytoplasm with increased intensity, suggesting up-regulation (Supplementary Figure S4). IL-6 and IL-17 staining in normal tissues was weak, reflecting immune homeostasis. In cases of obesity, IL-6 expression significantly increased, displaying dark brownish-yellow cytoplasmic staining, while IL-17 positivity rose, particularly in areas of interstitial inflammation, indicating progressive inflammation and expanded mediator production (Supplementary Figure S4). Concurrently, STAT3 phosphorylation levels were significantly elevated (Figure 4A and G), indicating activation of the IL-6/STAT3 signaling pathway.

In normal kidneys, ACE2 staining showed moderate positivity, with granular cytoplasmic and brush border staining in the proximal tubules. In contrast, ACE2 expression decreased in obese mice, exhibiting weaker positivity. Na/K-ATPase α1 in normal tissues exhibits moderate to strong linear basolateral membrane staining, which is essential for ion transport. However, in obesity, we observed reduced Na/K-ATPase α1 positivity and abnormal distribution, suggesting impaired tubular function (Supplementary Figure S4).

Collectively, these results demonstrate that obesity alters renal Na/K-ATPase signaling, inflammatory markers, HO-1, and ACE2 expression, thereby contributing to dysregulated sodium metabolism and reflecting the renal physiology and function associated with obesity. Notably, in normal mice, a high-salt diet activated both Na/K-ATPase and IL-6/p-STAT3 signaling pathways, while up-regulating HO-1 expression. These findings highlight the critical roles of these interconnected pathways and HO-1 in regulating systemic sodium homeostasis.

### CoPP regulates sodium metabolic pathways

Oxidative stress is a significant factor in the progression of kidney disease. In the kidney, physiological ROS acts as a secondary messenger that activates Na/K-ATPase signaling through a positive feedback mechanism. This process involves the phosphorylation of Src and ERK [20,28], which regulates the transport of Na^+^ in renal proximal tubules [6,28,29]. We hypothesized that the up-regulation of HO-1 may reduce oxidative amplification and inhibit the Na/K-ATPase signaling pathway, combined with the IL-6/p-STAT3 pathway to regulate renal sodium metabolism.

**Figure 5:**
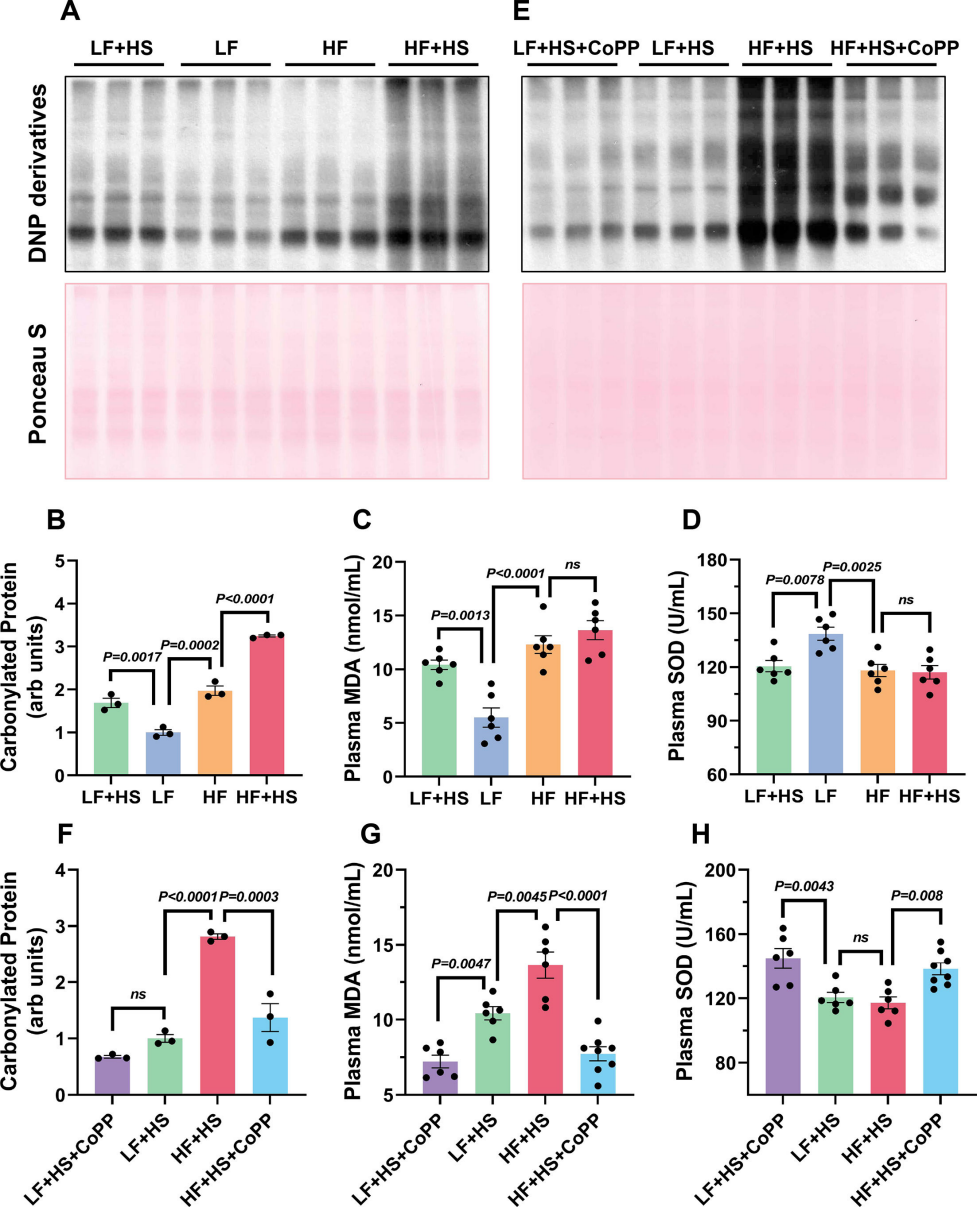
CoPP attenuates high salt-induced renal oxidative stress. **A, B, E, F**. Representative Western blot analysis of protein carbonylation in renal cortical homogenates with quantitative data (For protein carbonylation assay, the Ponceau S stained membrane was used for loading control.) **C,D**. The levels of MDA and SOD in plasma of control and obese mice after one week of high salt diet. **G,H**. The levels of MDA and SOD in plasma of control and obese mice after CoPP intervention. Data represent mean  ±  SEM; the two-way ANOVA with Tukey’s post hoc comparison was used for statistical significance. *n* = 3 in **B**,**F**. *n* = 6 default 8 in **C**,**D**,**G**,**H**. ^****^ *P*<0.001, ^***^ *P*<0.001, ^**^ *P*<0.01, ^*^ *P*<0.05, respectively.

To validate this hypothesis, we quantified systemic oxidative stress markers in plasma. The results indicated increased levels of MDA and protein carbonylation (Figure 5A–5C), along with decreased SOD during obesity (Figure 5D), suggesting enhanced renal oxidative stress. Treatment with CoPP resulted in reduced protein carbonylation and MDA levels while simultaneously increasing SOD levels (Figure 5E–5H). In conclusion, CoPP significantly alleviated oxidative stress induced by obesity.

Western blotting and IHC analyses revealed that CoPP treatment significantly up-regulated HO-1 expression in renal tissues (Figure 4B and H **, S4**). However, the regulation of ACE2 expression by CoPP was context-dependent: it down-regulated ACE2 in normal mice under high-salt diet but up-regulated it in obese mice, while Western blot analysis did not show statistically significant changes in overall ACE2 protein levels (Figure 4B and I). CoPP reduced pro-inflammatory mediators (IL-6 and IL-17, Supplementary Figure S4) and suppressed both Na/K-ATPase and IL-6/p-STAT3 signaling, as evidenced by decreased phosphorylation of Src, ERK1/2, and STAT3 (Figure 4B and J–L). These findings further support our conclusion that HO-1 regulates the Na/K-ATPase signaling pathway by alleviating oxidative stress, thereby exerting bidirectional regulatory effects on renal sodium metabolism. In normal mice, HO-1 inhibits sodium excretion by suppressing Na/K-ATPase signaling, whereas in obese mice, HO-1 modulates renal sodium metabolism by restoring homeostasis of the constitutively activated Na/K-ATPase signaling pathway.

### Effect of cardiotonic steroids on oxidative stress and signaling in LLC-PK1 and HK-2 cells

Cardiotonic steroids (CTS), as endogenous ligands for the Na/K-ATPase, activate c-Src-mediated signaling pathways (including ERK1/2, PI3K, PKC, and ROS production) through binding to the α1 subunit, thereby regulating renal sodium metabolism and contributing to the pathogenesis of salt-sensitive hypertension [30]. Endogenous CTS levels increase in response to impaired renal sodium excretion or high salt intake [31], thereby influencing renal Na^+^ excretion and blood pressure through Na/K-ATPase signaling pathway [32].

In this study, two typical CTS, ouabain (Ouab) and digoxin (Dig), were selected to induce Na/K-ATPase signaling activation and oxidative stress amplification *in vitro*. We used CTS concentrations known to activate Na/K-ATPase signaling: 100 nM for LLC-PK1 cells and 10 nM for HK-2 cells [28]. Through CoPP pretreatment, we aimed to determine whether HO-1 up-regulation participates in regulating Na/K-ATPase signal transduction and specifically blocks oxidative amplification. The dose and duration of CoPP pretreatment were determined based on the extent of HO-1 up-regulation induced by CoPP (Supplementary Figure S5 and S6). As assessed by the CCK-8 assay (Supplementary Figure S7), these concentrations did not significantly affect cell viability.

**Figure 6:**
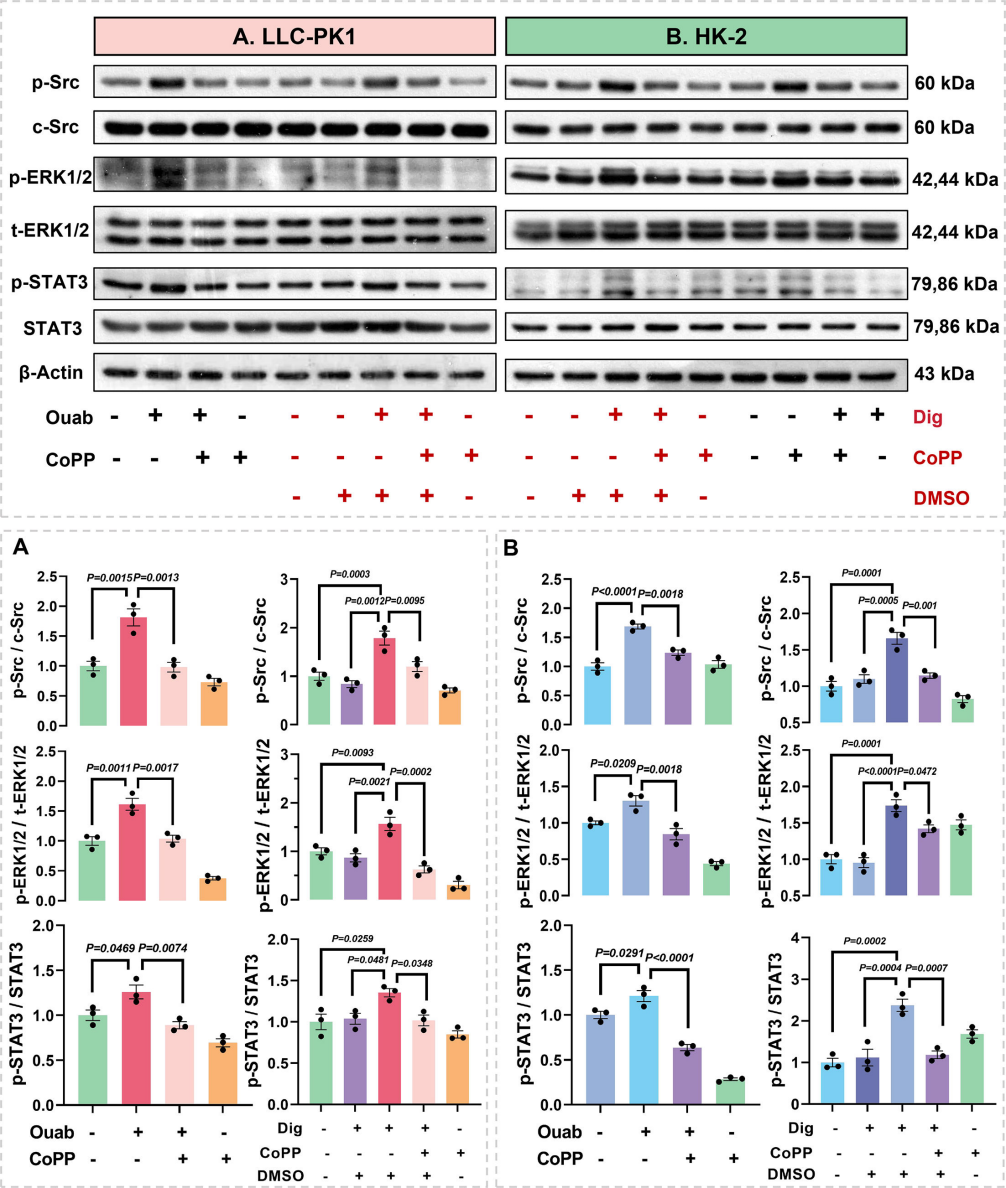
Effects of CoPP pretreatment on CTS-induced signal transduction in LLC-PK1 and HK-2 cells. **(A**) LLC-PK1 and (**B**) HK-2 cells were used for these *in vitro* studies demonstrating the effects of CoPP on ouabain or digoxin induced Src activation, ERK1/2 activation, and STAT3 activation. Data represent mean  ±  SEM; the one-way ANOVA with Tukey’s post hoc comparison was used for statistical significance. *n* = 3. ^****^ *P*<0.001, ^***^ *P*<0.001, ^**^ *P*<0.01, ^*^ *P*<0.05, respectively.

**Figure 7:**
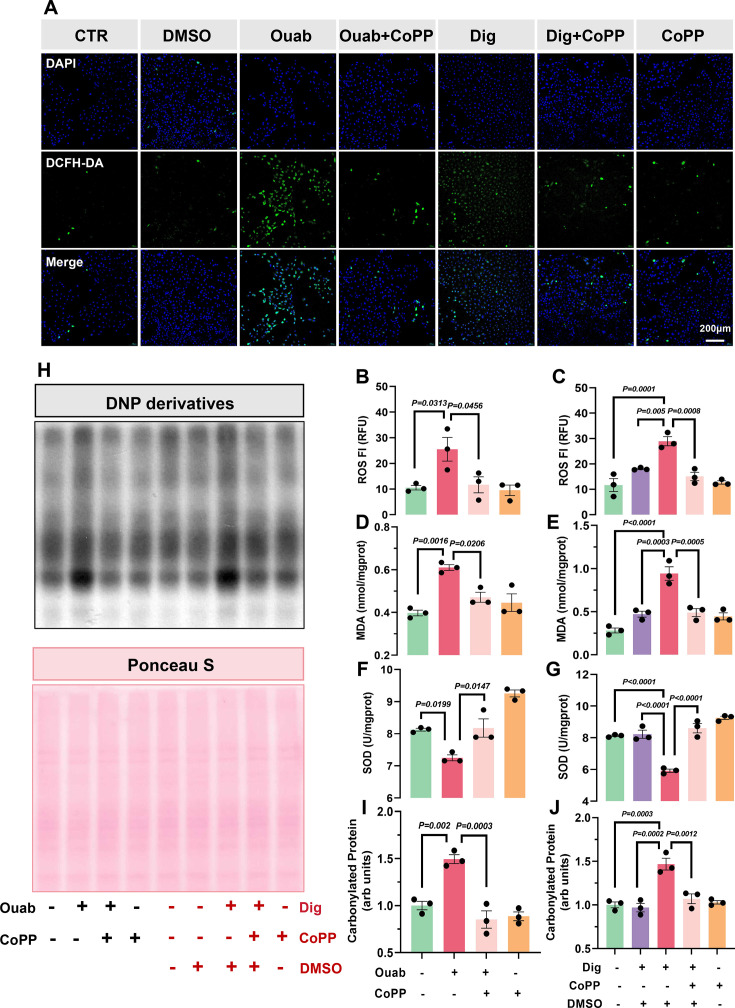
CoPP attenuates CTS-induced oxidative stress in LLC-PK1 cells. **A**. Representative fluorescent images showing intracellular ROS levels detected by the DCFH-DA probe. **B, C** quantitative analysis of ROS fluorescence intensity (FI) measured using a microplate reader. **D-G**. MDA and SOD levels in cell lysates under different treatments. **H-J**. Western blot analysis of protein carbonylation with quantification. Data represent mean  ±  SEM; the one-way ANOVA with Tukey’s post hoc comparison was used for statistical significance. *n* = 3. ^****^ *P*<0.001, ^***^ *P*<0.001, ^**^ *P*<0.01, ^*^ *P*<0.05, respectively.

The results showed that Ouab and Dig activated the Na/K-ATPase signaling pathway in LLC-PK1 and HK-2 cells, as evidenced by increased phosphorylation of Src and ERK1/2, while also activating STAT3. HO-1 up-regulation significantly inhibited the activation of these pathways (Figure 6A and B). Furthermore, Ouab and Dig significantly increased ROS levels (Figure 7A–7C **, S8**), MDA content (Figure 7D and E), and protein carbonylation (Figure 7H–7J), while decreasing SOD levels in LLC-PK1 cells (Figure 7F and G), indicating that CTS induces oxidative stress amplification *in vitro*. CoPP pretreatment effectively reduced CTS-induced ROS, protein carbonylation, and MDA levels, while also enhancing SOD levels (Figure 7A–7J **, S8**). Thus, CoPP significantly mitigated oxidative stress induced by CTS. Similar results were noted in HK-2 cells (Supplementary Figure S9 and S10).

Furthermore, we treated LLC-PK1 cells with IL-6 following CoPP pretreatment. The findings showed that IL-6 also activated ERK1/2 and STAT3 pathways in LLC-PK1. CoPP attenuated this activation (Supplementary Figure S11), suggesting that an interconnection between these pathways and their potential cross-activation by common upstream signaling molecules, such as CTS or IL-6. CoPP treatment effectively suppressed the activated p-STAT3 and p-ERK1/2 signaling pathways to normal levels.

### Obesity or CoPP-induced gene expression alterations in the kidney

**Figure 8:**
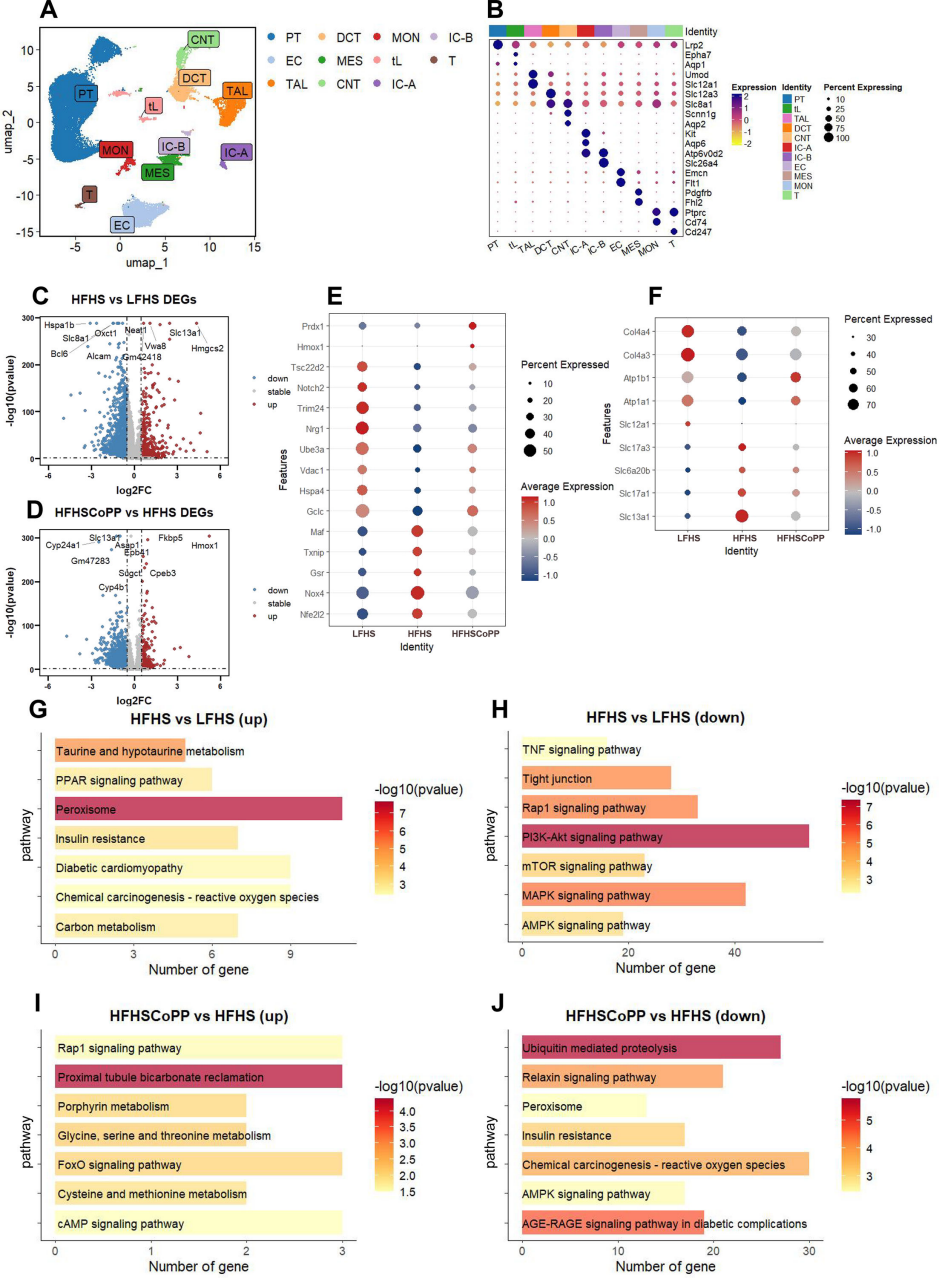
Single-nucleus RNA sequencing reveals cell-type-specific responses and transcriptomic alterations in mouse kidney under high-fat high-salt diet and CoPP treatment. **A**. Integration and clustering of kidney snRNA-seq data from LF + HS, HF + HS, and HF + HS default CoPP groups. **B**. Cell type identification based on established renal cell lineage-specific marker genes. **C-D**. Volcano plots displaying differentially expressed genes (DEGs) between groups: (**C**) HFHS vs. LFHS; (**D**) HFHS + CoPP vs. HFHS. Down-regulated and up-regulated genes are shown in blue and red, respectively. **E**. Expression changes of oxidative stress-related genes after CoPP treatment. **F**. Alterations in gene expression associated with sodium and potassium ion metabolism following CoPP administration. **G-J**. Enriched KEGG pathways among up- and down-regulated DEGs affected by HF + HS diet and CoPP treatment. CNT, connecting tubule; DCT, distal convoluted tubule; EC, endothelial cell; IC-A, type A intercalated cell; IC-B, type B intercalated cell; MES, mesangial cell; MON, monocyte; PT, proximal tubule; TAL, thick ascending limb; tL, thin limb of Henle’s loop.

To further explore the transcriptomic changes induced by CoPP treatment, we performed single-nucleus RNA sequencing (snRNA-seq) on left kidney tissues from three experimental groups: LF + HS, HF + HS, and HF + HS default CoPP mice. Data integration, dimensionality reduction, and clustering were conducted using Seurat. The graph-based clustering reveals distinct populations of major renal cell types, which were annotated based on established marker genes, including proximal tubule (PT) cells, distal convoluted tubule (DCT) cells, and thick ascending limb (TAL) cells, among others (Figure 8A and B).

Having established this cell type-resolved transcriptional landscape, we next sought to identify specific gene expression alterations and functional pathways associated with dietary intervention and CoPP treatment. Differential gene expression and KEGG Enrichment Analysis were performed between each of the three groups. Obese mice on a high-salt diet had increased expression of the sodium-sulfate cotransporter, Slc13a1, and had down-regulated expression of Slc8a1 encoding the sodium-calcium exchanger [33] (Figure 8C). The up-regulation of Slc13a1 may reflect compensatory mechanisms in response to high-salt stress [34]. CoPP may modulate renal sodium metabolism and function by up-regulating Hmox1 and down-regulating Slc13a1 (Figure 8D).

There were significant dysregulations of certain genes that regulate oxidative stress and sodium metabolism in the renal cells of obese mice on a high-salt diet. Key oxidative stress-related genes, including Nfe2l2, Nox4, Gsr, Txnip, Maf, were up-regulated. In contrast, genes involved in antioxidant defense (Gclc, Hspa4, Vdac1, Ube3a) and cellular repair (Nrg1, Trim24, Notch2, Tsc22d2) were down-regulated (Figure 8E). This pattern indicates that obesity exacerbates oxidative stress and compromises the kidney’s capacity to counteract damage. Furthermore, obesity led to the up-regulation of sodium transporters (Slc13a1, Slc22a8, Slc17a1, Slc17a3) and the down-regulation of structural proteins (Col4a3, Col4a4) as well as Na/K-ATPase subunits encoded by Atp1a1, Atp1b1, suggesting impaired sodium metabolism and underlying renal injury (Figure 8F). CoPP partially ameliorated these alterations by down-regulating oxidative stress-related genes and up-regulating key antioxidant and repair genes implicated in protection against oxidative damage. Furthermore, CoPP restored the expression of sodium transporters through the up-regulation of Slc12a1, Atp1a1, and Atp1b1. These alterations are anticipated to enhance sodium homeostasis and restore renal integrity, thereby facilitating the recovery of renal function.

On a high-salt diet, obese mice demonstrated significant metabolic and signaling dysregulation. Key pathways such as the MAPK and AMPK signaling pathways were down-regulated (Figure 8H), suggesting impaired cellular stress responses and energy homeostasis. Conversely, peroxisomes, insulin resistance, and chemical carcinogenesis - reactive oxygen species pathways were up-regulated (Figure 8G), reflecting elevated oxidative stress and metabolic dysfunction. Obesity-induced oxidative stress may drive sustained activation of the Na/K-ATPase signaling pathway (the phosphorylation of ERK1/2, Figure 4A), ultimately leading to the pathway desensitization, and down-regulation of the MAPK signaling pathway may be an adaptive response to this phenomenon, which is one of the underlying mechanisms contributing to the impaired sodium excretion observed in obese mice under high salt intake. CoPP treatment effectively mitigated these alterations by up-regulating proximal tubular bicarbonate recycling and FoxO signaling pathway (Figure 8I), thereby restoring sodium and acid-base homeostasis. Additionally, CoPP down-regulated insulin resistance and chemical carcinogenesis - reactive oxygen species (Figure 8J), thereby reducing oxidative stress. Furthermore, CoPP restored Rap1 and cAMP signaling pathways, enhancing cellular communication and stress adaptation.

### Metabolic profiling alterations in obese mice treated with CoPP

**Figure 9:**
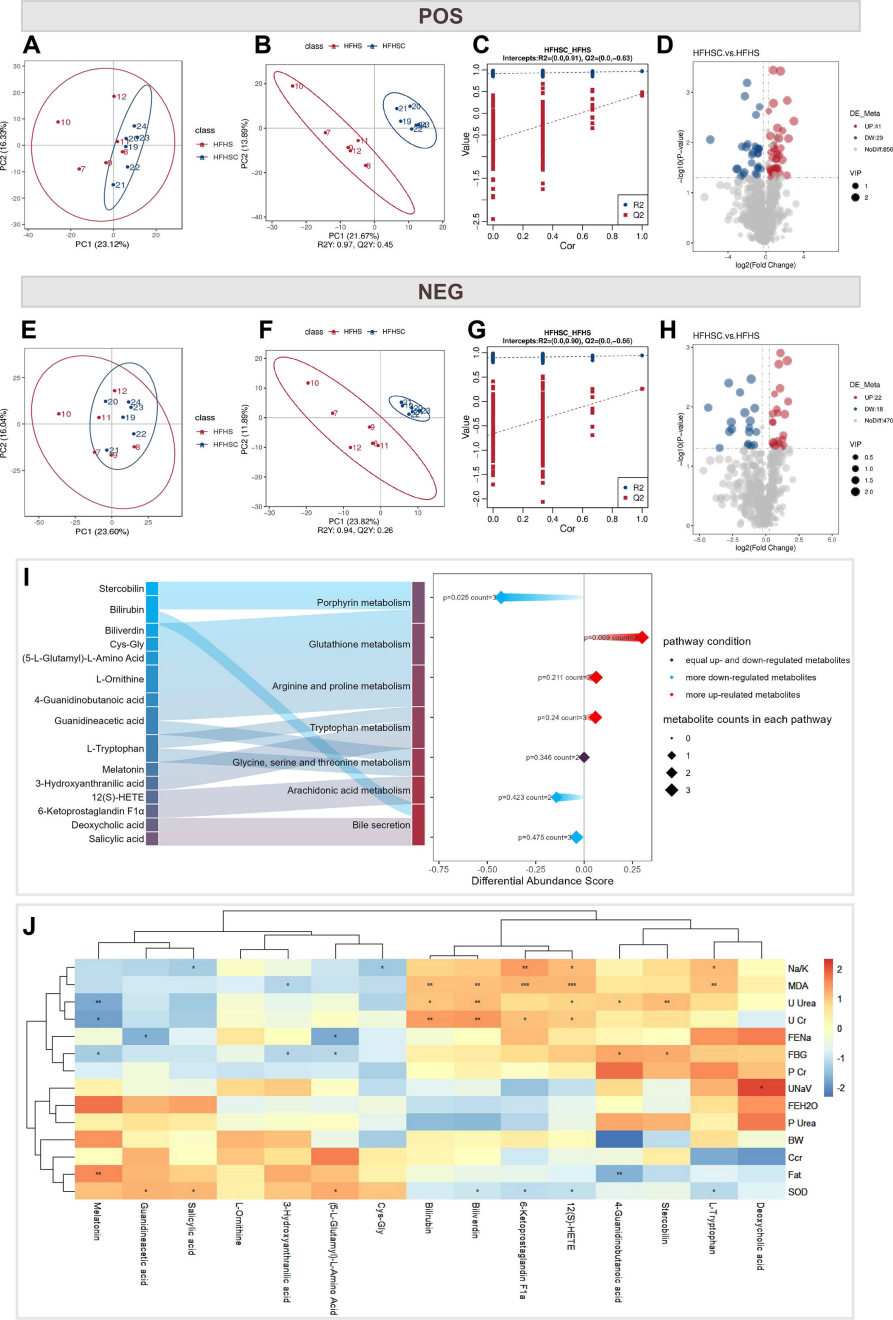
Overview of the metabolic profiling alterations in obese mice treated with the CoPP. **A,E**.PCA plots of urine-specific samples in positive (POS) and negative (NEG) ion modes, respectively. **B,F**.PLS-DA plots of urine-specific samples in POS and NEG ion modes respectively. The two groups were well separated in the PLS-DA score plot, indicating that they had markedly different metabolic characteristics. **C,G**. 200 permutation tests for the PLS-DA models. All the red regression lines of the Q2 points intersect the vertical axis at values less than 0 indicating the excellent reliability, predictability and no over-fitting for the PLS-DA model. **D,H**.Volcano plots of all the detected metabolites in the urine samples in POS and NEG ion modes, respectively. Red represents up-regulated, and blue represents down-regulated; **I**. Pathway enrichment analysis of differential metabolites with KEGG database analysis. **J**.Heatmap analysis of differential metabolites correlated with obesity, oxidative stress and renal function indicators

To investigate the role of HO-1 in renal sodium metabolism, we analyzed 24 hour urine samples from mice subjected to high-fat and high-salt diets (HF + HS) and those treated with CoPP (HF + HS default CoPP) (*n* = 6) using non-targeted metabolomics. In total, 510 metabolites were identified in negative ion mode, and 926 were identified in positive ion mode, using the HMDB, Lipidmaps, KEGG, and MassList databases. Principal component analysis (PCA) revealed high clustering in both ion modes, demonstrating data homogeneity (Figure 9A and E). PLS-DA showed clear separation between the two groups in both modes (Figure 9B and F). Permutation tests (*n* = 200) confirmed the robustness of the PLS-DA model, yielding R^2^/Q^2^ values of 0.91/–0.63 for the positive mode and 0.90/–0.66 for the negative mode, indicating model stability, reliability, and predictability without overfitting (Figure 9C and G). These findings highlight significant differences in urinary metabolites between HF + HS and HF + HS default CoPP mice.

Volcanogram analysis uncovered potential differential metabolites using the following thresholds of VIP > 1.0, FC > 1.2 or < 0.833, and P-value < 0.05 (Figure 9D and H). A total of 40 were identified in negative ion mode, and 70 differential metabolites in positive ion modes, with the top ten metabolites listed in Supplementary Table S3. KEGG enrichment analysis revealed that metabolic pathways significantly affected by HO-1 up-regulation were primarily related to amino acid metabolism (Supplementary Figure S12 and S13). These differential metabolites were associated with 36 KEGG pathways (Supplementary Table S4), with porphyrin metabolism showing significant enrichment (*P*<0.05). These findings highlight key metabolic and signaling pathways influenced by CoPP treatment.

Through mulberry diagram analysis, we identified seven oxidative stress-related metabolic pathways involving 15 metabolites (Figure 9I, Supplementary Table S5). Urinary bilirubin, biliverdin [13], and stercobilin levels decreased after CoPP treatment, likely due to increased cellular retention for antioxidant defense [35]. They are three porphyrin metabolites, contribute to metabolic homeostasis and exhibit antioxidant properties [36]. CoPP also enhances glutathione (GSH) metabolism, elevating metabolites like Cys-Gly and (5-L-Glutamyl)-L-Amino Acid, indicating increased GSH activity in response to oxidative stress [37]. Additionally, CoPP influences amino acid metabolism, affecting arginine, L-ornithine, and L-tryptophan pathways, which may affect GSH synthesis and redox balance [38,39]. Melatonin, a tryptophan-derived antioxidant, may further regulate tryptophan metabolism [40]. Arachidonic acid metabolism is interfered with by COPP, which inhibits the excretion of 6-Ketoprostaglandin Fla and 12(S)-HETE. 6-Ketoprostaglandin Fla exhibits renal vasodilatory and antioxidant effects [41], while 12(S)-HETE promotes inflammation and oxidative stress [42]. CoPP also reduces urinary excretion of deoxycholic acid, which may restore function of bile acids [43].

Correlation analysis of metabolites with obesity parameters revealed that 4-Guanidinobutanoic acid showed a significant negative correlation with fat weight (*r* = –0.74, **P<0.01). At the same time, melatonin exhibited a significant positive correlation with fat weight (*r* = 0.72, **P<0.01), suggesting its potential roles in adipogenesis regulation. Bilirubin, biliverdin, 6-ketoprostaglandin F1α, and 12(S)-HETE demonstrated significant positive correlations with MDA, whereas biliverdin, 6-ketoprostaglandin F1α, and 12(S)-HETE showed significant negative correlations with SOD. This suggests that CoPP might ameliorate oxidative stress by modulating these metabolites, thereby alleviating metabolic disorders. Furthermore, bilirubin, biliverdin, 6-ketoprostaglandin F1α, and 12(S)-HETE were positively correlated with urinary creatinine, while bilirubin, biliverdin, stercobilin, 4-guanidinobutanoic acid, and 12(S)-HETE were positively correlated with blood urea nitrogen, suggesting that CoPP-induced enhancement of heme metabolism and arachidonic acid metabolism might be involved in renal function regulation. Additionally, 6-ketoprostaglandin F1α, 12(S)-HETE, and L-tryptophan exhibited positive correlations with the Na^+^/K^+^, whereas guanidinoacetic acid and (5-L-glutamyl)-L-amino acid showed negative correlations with FENa^+^, implying that CoPP might interfere with renal sodium metabolism through modulation of these metabolites (Figure 9J, Supplementary Table S6).

Overall, the induction of HO-1 affects multiple metabolic pathways, including porphyrin metabolism, glutathione metabolism, and amino acid metabolism. These pathways are crucial for counteracting oxidative stress, improving metabolic homeostasis, and regulating renal sodium metabolism. The present study also reveals changes in specific metabolites and their association with obesity, oxidative stress, and renal sodium metabolism under CoPP intervention. These findings provide valuable insights into the mechanisms by which HO-1 regulates renal sodium metabolism and salt-sensitive hypertension.

## Discussion

Obese mice exhibited impaired sodium metabolism under high-salt diet conditions, accompanied by up-regulated HO-1 expression, down-regulated ACE2 expression, and enhanced oxidative stress. In normal mice, high-salt intake activated the renal Na/K-ATPase signaling pathway (as evidenced by Src and ERK1/2 phosphorylation), whereas obese mice showed elevated basal activity of this pathway but failed to respond to high-salt stimulation, suggesting impaired signal transduction. These results demonstrate that obesity disrupts renal sodium metabolism through co-ordinated alterations in Na/K-ATPase signaling, oxidative stress, HO-1, and ACE2 expression. Notably, the salt-responsive activation of both Na/K-ATPase signaling and oxidative stress pathways, along with HO-1 up-regulation, collectively constitutes a critical regulatory network for systemic sodium homeostasis.

In this study, we demonstrated that HO-1 regulates renal sodium metabolism by modulating the Na/K-ATPase signaling pathway and its associated oxidative stress through CoPP treatment in mice under high-salt diet conditions. Our findings reveal that HO-1 exerts its effects via two main mechanisms: (1) attenuating oxidative stress and inflammatory damage by suppressing protein carbonylation, reducing MDA levels, and decreasing inflammatory cytokines; (2) improving sodium metabolism through regulation of Na/K-ATPase signaling—inhibiting this pathway to reduce sodium excretion in normal mice, while restoring normal function of the constitutively activated pathway in obese mice. This finding reveals the complex regulatory role of HO-1 in sodium metabolism.

**Figure 10:**
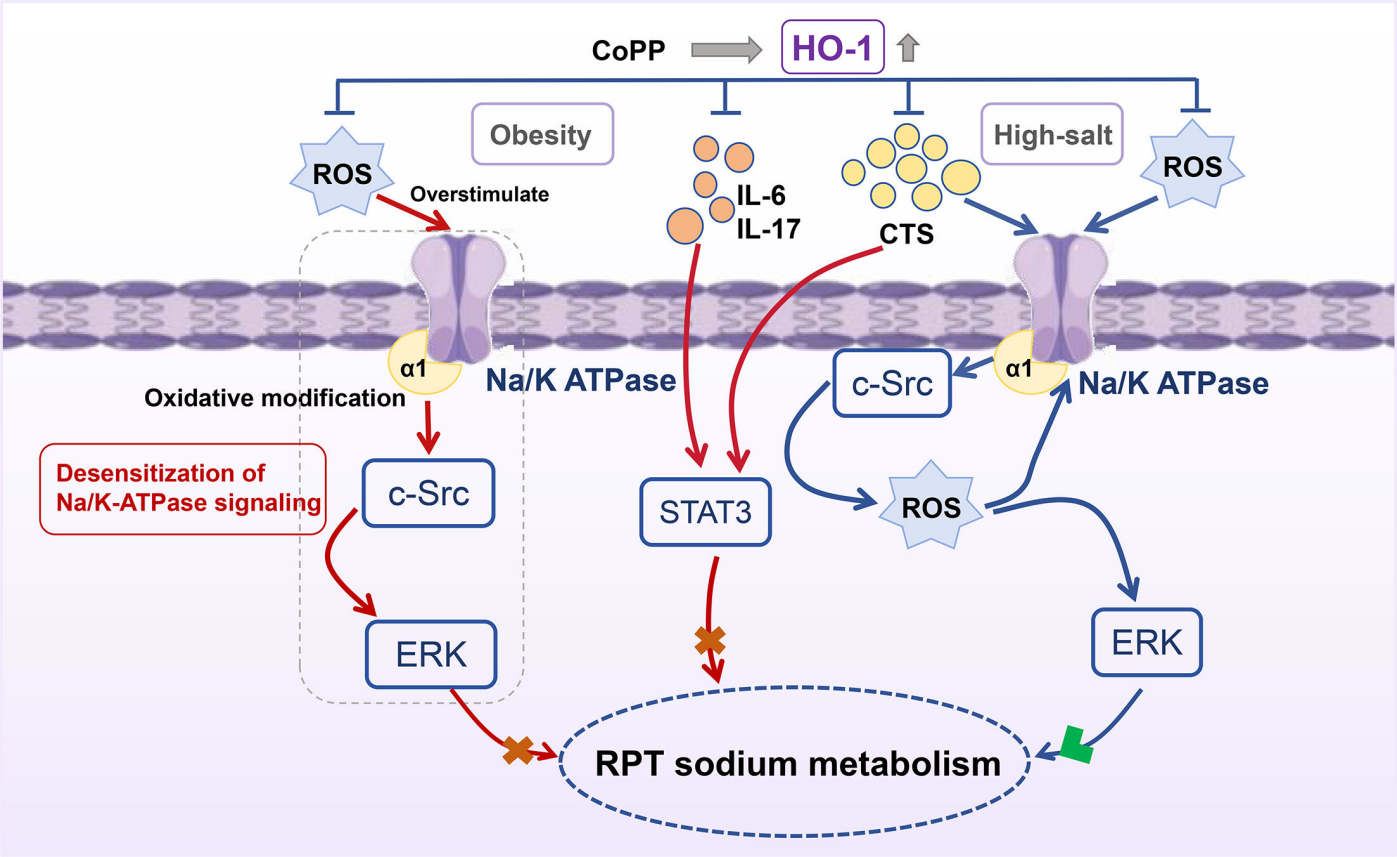
Mechanistic diagram of CoPP regulating renal sodium metabolism.

Endogenous CTS levels rise in response to impaired renal sodium excretion or high salt intake [31]. The CTS-induced activation of the Na/K-ATPase signaling pathway amplifies ROS production and enhances downstream signal transduction, establishing a positive feedback loop. Notably, although HO-1 induction significantly reduced oxidative stress levels, the consequent decrease in ROS may fail to further activate Na/K-ATPase signaling, thereby limiting sodium excretion. In obese mice, CTS-induced ROS is essential for signaling that inhibits sodium reabsorption in RPT [44]. However, prolonged stimulation of ROS may lead to oxidative modification of the Na/K-ATPase α1 subunit, desensitizing the signaling pathway and potentially causing an imbalance in sodium reabsorption and metabolism [6]. HO-1 regulation of oxidative stress and the Na/K-ATPase signaling pathway may potentially restore the sensitivity of this pathway, thereby benefiting the recovery of renal sodium metabolic homeostasis (Figure 10). Together, these findings provide important implications for understanding the complex relationship among CTS levels, oxidative stress, and sodium excretion and could potentially lead to new strategies for managing sodium balance in conditions such as obesity.

Therefore, the induction of HO-1 presents a double-edged sword. While CoPP effectively combats oxidative stress, the short-term induction of HO-1 inhibits ROS production, negatively affecting sodium excretion. Conversely, excessive ROS stimulation over time can desensitize the Na/K-ATPase signaling pathway and disrupt sodium metabolism. The timing of sodium excretion facilitated by HO-1 or the identification of new, effective antioxidants may restore the sensitivity of the Na/K-ATPase signaling pathway, which warrants further research to understand the paradoxical nature of HO-1 induction fully.

Furthermore, both genetic analysis and urinary metabolite assessment have more recently confirmed the regulatory effects of CoPP on oxidative stress and sodium metabolism. CoPP improves obesity-induced renal sodium metabolism disorders by modulating oxidative stress genes and restoring sodium transporters. It counteracts obesity-induced metabolic dysregulation by enhancing antioxidant defense and renal tubular repair mechanisms, thereby improving sodium homeostasis and renal integrity. The present study also revealed changes in specific metabolites and their correlation with obesity, oxidative stress, and renal sodium metabolism under CoPP intervention, as well as the induction of HO-1, which affects multiple metabolic pathways, including porphyrin metabolism, glutathione metabolism, and amino acid metabolism. These findings offer valuable insights into how HO-1 regulates renal sodium metabolism and contributes to salt-sensitive hypertension.

The role of HO-1 in regulating renal sodium metabolism and its potential impact on salt-sensitive hypertension opens new avenues for therapeutic interventions. The dual nature of HO-1, acting as both a defender against oxidative stress and a regulator of sodium excretion, highlights the complexity of its functions within the body. There is a pressing need for future research to delve into the precise mechanisms by which HO-1 modulates the Na/K-ATPase signaling pathway. Future studies will also incorporate direct hemodynamic measurements, including blood pressure monitoring, to more comprehensively evaluate the physiological outcomes of HO-1 induction. Identifying the optimal conditions for its induction to balance oxidative stress reduction with effective sodium excretion is crucial. Additionally, exploring novel antioxidants that complement HO-1’s protective effects without compromising sodium metabolism could lead to innovative treatments for obesity-related hypertension. The insights gained from this study provide a solid foundation for developing targeted therapies that leverage the benefits of HO-1 up-regulation while mitigating its potential drawbacks, ultimately contributing to improved management of hypertension and ROS-related metabolic disorders.

## Conclusion

Our study demonstrates that HO-1 regulates renal sodium metabolism through two key mechanisms: attenuating oxidative stress and inflammation and modulating Na/K-ATPase signaling pathway. Although HO-1 reduces oxidative stress, the consequent decrease in ROS may impair Na/K-ATPase signaling and sodium excretion. Conversely, chronic oxidative stress desensitizes the Na/K-ATPase signaling pathway, exacerbating sodium imbalance. Under these conditions, HO-1-mediated suppression of oxidative stress may potentially restore pathway sensitivity, thereby promoting the recovery of renal sodium metabolic homeostasis. Integrated genetic and metabolomic analyses reveal that HO-1 reprograms oxidative stress responses, sodium transporter activity, and critical metabolic pathways (e.g., porphyrin, glutathione, and amino acid metabolism). These findings establish HO-1 as a central regulator linking oxidative stress, sodium handling, and metabolic dysfunction in obesity. Our work provides a novel perspective on HO-1’s dual role in sodium metabolism and highlights its therapeutic potential for obesity-related salt-sensitive hypertension, though precise modulation of its redox-dependent effects remains a challenge for future translational research.

**Clinical Perspectives**

- Rationale: Impairment of renal sodium metabolism caused by obesity is a significant factor contributing to the development of salt-sensitive hypertension. This study examined how obesity disrupts the kidneys’ ability to handle sodium through mechanisms involving oxidative stress and dysfunction in Na/K-ATPase signaling, with a particular focus on the dual role of HO-1 as both an antioxidant and a regulator of sodium metabolism.
- Key findings: Induction of HO-1 by CoPP reduced oxidative damage and regulated Na/K-ATPase sensitivity in obese mice. However, it is important to note that this induction also limited sodium excretion in normal mice by inhibiting the ROS-mediated Na/K-ATPase signaling pathway, demonstrating a complex interaction.
- Translational impact: These insights into the underlying mechanisms highlight HO-1 as a potential therapeutic target for salt-sensitive hypertension in individuals with metabolic disorders. Nevertheless, its activation needs to be carefully timed and optimized to successfully balance redox status and sodium homeostasis.

## Supplementary material

Online supplementary material 1

Online supplementary table 1

Online supplementary table 2

Online supplementary table 3

Online supplementary table 4

## Data Availability

All data generated or analyzed during this study are included in this published article and its supplementary information files. Additional datasets are available from the corresponding author upon reasonable request.

## Open Access

This article has been published open access under our Subscribe to Open programme, made possible through the support of our subscribing institutions, learn more here: https://portlandpress.com/pages/open_access_options_and_prices#conditional

## Abbreviations

ACE2: Angiotensin-converting enzyme 2
AFI: Abdominal fat index
BUN: Blood Urea Nitrogen
CRE: Creatinine
CTS: Cardiotonic steroids
Ccr: Creatinine clearance
CoPP: Co(III) Protoporphyrin IX chloride
Dig: Digoxin
ERK: Extracellular signal-regulated kinase
FBG: Fasting blood glucose
FEH2O: Fractional H2O excretion rate
FENa+: Fractional Na + excretion rate
GFR: Glomerular filtration rate
GSH: Glutathione
HF: High-fat diet
HO-1: Heme Oxygenase-1
HS: High-salt diet
IHC: Immunohistochemical staining
LF: Low-fat diet
MDA: Malondialdehyde
Na+/K+: the ratio of urinary excreted Na + to K + concentration
Ouab: Ouabain
PCA: Principal component analysis
PCr: Plasma creatinine
PI3K: Phosphoinositide-3 kinase
PKC: Protein kinase C
PLS-DA: Partial least squares discriminant analysis
RAAS: Renin-angiotensin-aldosterone system
ROS: Reactive oxygen species
SOD: Superoxide Dismutase
STAT3: Signal transducer and activator of transcription 3
TL: The length of the right tibia
UCr: Urinary creatinine
Urea: Plasma Urea Nitrogen
Urea: Urinary Urea Nitrogen
V: Urinary Na + excretion rate
c-Src: Cellular Src
