# Supplementary material for: HO-1 modulates obesity-related renal sodium metabolism via oxidative stress and Na/K-ATPase signaling
Source: Clin Sci (Lond). 2025 Nov 4;139(21):1301–20. doi: 10.1042/CS20257602 (PMC12687461; doi:10.1042/CS20257602)
Supplement: Online supplementary material 1 [file cs-139-21-CS20257602-s001.docx]

**Supporting Information**

**HO-1 modulates obesity-related renal sodium metabolism via oxidative stress and Na/K-ATPase signaling**

Jiahui Cai^1^, Feifei Sun^1^, Qiaoyun Pan^1^, Shasha Zhao^1^, Yunbo Sun^1^, Feng Yang^1^, Danshu Wang^1^, Runyan Tan^1^, Weiping Liu^2^, Qiang Tan^3^, Xue Shao^2^, Sandrine V. Pierre^4^ and Yanling Yan^*1,5^

^1^Key Labs Nanobiotech & Applied Chemistry, Department of Biotechnology & Engineering, College of Environmental & Chemistry Engineering, Yanshan University, Qinhuangdao, 066004, China.

^2^Divisions of Nephrology & ^3^Cardiology, The First Hospital of Qinhuangdao, Qinhuangdao, China.

^4^Department of Biomedical Sciences, ^5^Joan C. Edwards School of Medicine, Marshall University, Huntington, WV 25755, U.S.A.

**Corresponding author at: Department of Biological Engineering, College of Environment & Chemical Engineering, Yanshan University, No.438 Hebei street, Qinhuangdao 066004, P.R. China*

*Tel + 13273353286; Email address: yanyanl@ysu.edu.cn (Yanling Yan)*

**Supporting results**

**Figure S1**. Body weight change curves for C57BL/6J mice in the control (low-fat diet, LF) and high-fat diet (HF) groups.

**Figure S2.** Body weight and Fasting blood glucose (FBG) in control and obese mice after 1-week high-salt diet and CoPP intervention. (Data represent mean ± SEM; the two-way ANOVA with Tukey’s post-hoc comparison was used for statistical significance. The red highlighting represents the overall difference between the control and obese mouse groups. n=6~8. ^****^*P*<0.001, ^***^*P*<0.001, ^**^*P*<0.01, ^*^*P*<0.05, respectively.)

**Figure S3**. Urinary Na^+^ output/Na^+^ intake ratio, urine output/water intake ratio, urinary Na^+^ excretion rate (UNa^+^V), fractional Na^+^ excretion (FENa^+^), and fractional water excretion (FEH_2_O), and urinary Na^+^/K^+^ in control and obese mice after 1-week high-salt diet. (The data were expressed as the means±SD. The statistical significance was calculated using the unpaired, two-tailed, Student’s t-test. n=6. *****P*<0.001, ****P*<0.001, respectively.)

**
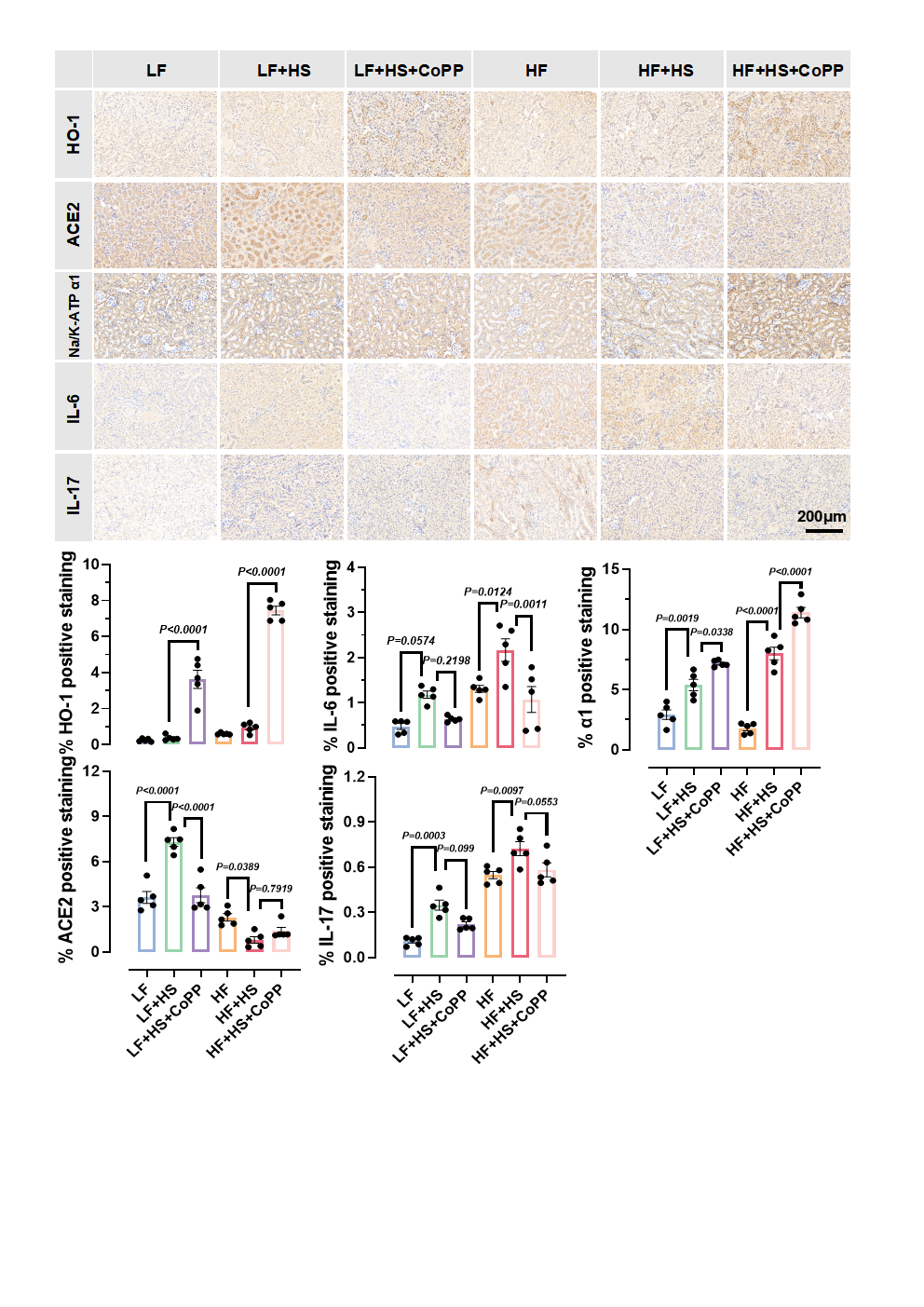
**

**Figure S4**. Representative immunohistochemical staining of HO-1, Na/K-ATPase α1, IL-6, IL-17, and ACE2 in kidney tissue sections with quantitative analysis performed using ImageJ software. (Data represent mean ± SEM; the two-way ANOVA with Tukey’s post-hoc comparison was used for statistical significance. n=5. ^****^*P*<0.001, ^***^*P*<0.001, ^**^*P*<0.01, ^*^*P*<0.05, respectively.)

**HO-1**

**β-Actin**

**LLC-PK1**

**43kDa**

**32kDa**


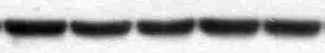

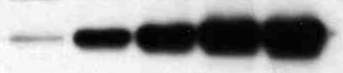


**HO-1**

**β-Actin**

**CTR 0.5 1 2 3 (μM)**

**CoPP, 24h**

**HK-2**

**32kDa**

**43kDa**


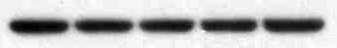

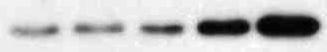


**Figure S5**. With the increase of CoPP concentration, HO-1 expression increased in LLC-PK1 and HK-2 cells,and a dose of 2uM was selected for cell pretreatment.(The data were expressed as the means±SD, n=3)


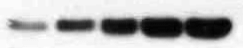


**CTR 6 12 24 48 (h)**

**CoPP, 2μM**


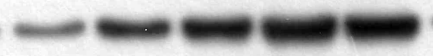

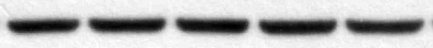


**HO-1**

**β-Actin**

**32kDa**

**43kDa**

**HO-1**

**β-Actin**

**32kDa**

**43kDa**

**LLC-PK1**

**HK-2**


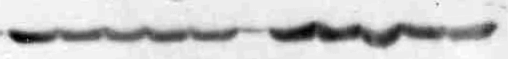


**Figure S6**. With the prolongation of CoPP treatment time, HO-1 expression increased in LLC-PK1 and HK-2 cells, and 24 h was selected as the cell pretreatment time.(The data were expressed as the means±SD, n=3)

**Figure S7.** Cell viability of LLC-PK1 and HK-2 at different treatment modalities by CCK8 assay. (For LLC-PK1 cells: Ouabain,0.1μM,1h; Digoxin,0.1μM,1h; CoPP:2μM,24h. For HK-2 cells: Ouabain,10nM,1h; Digoxin,10nM,1h; CoPP:2μM,24h).The results showed that all the above treatments had no significant effect on cell viability.(The data were expressed as the means±SD, The statistical significance was calculated using the unpaired, two-tailed, Student’s t-test. n=5. ns, not significant.)


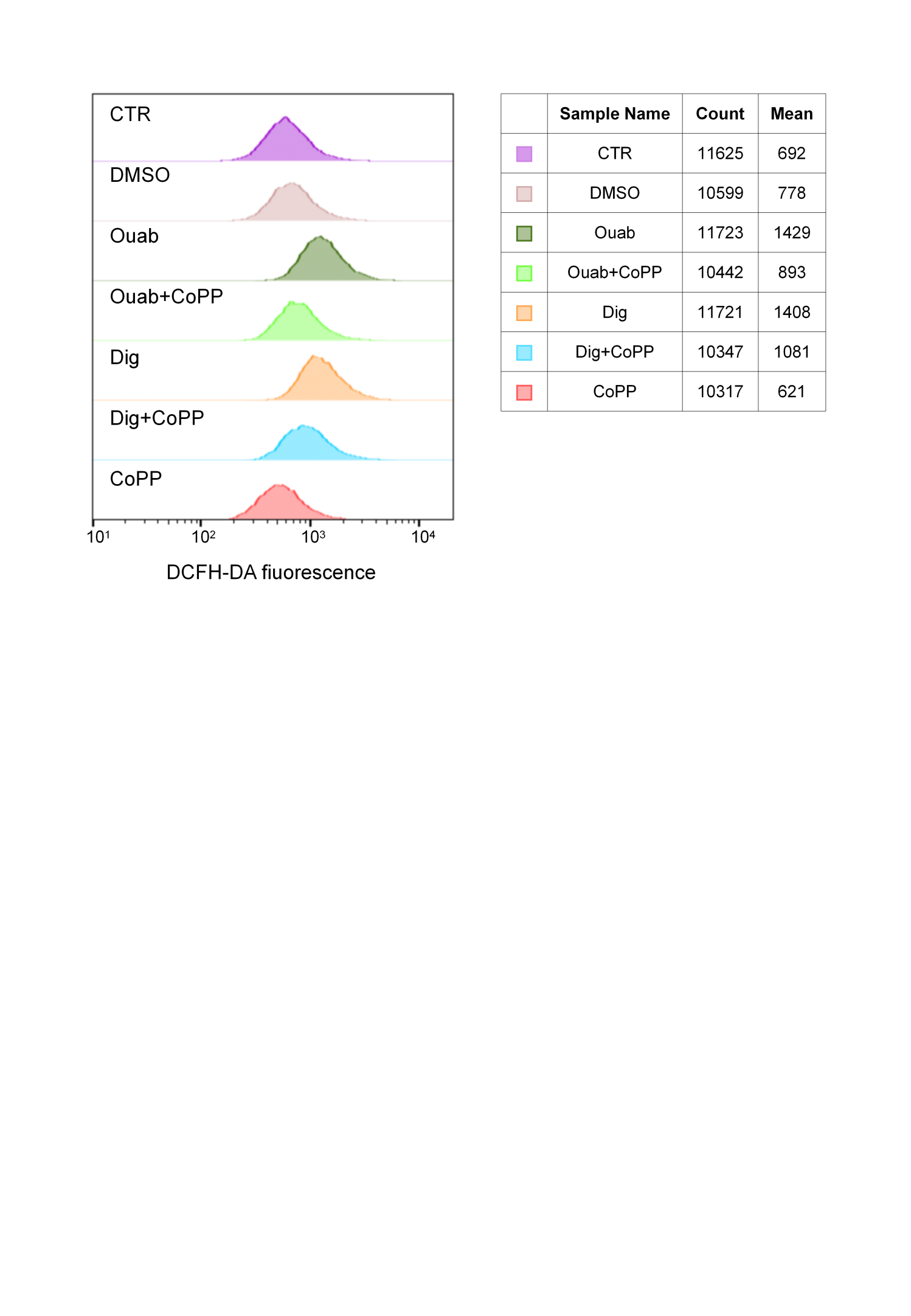


**Figure S8.** The ROS levels in LLC-PK1 cells treated with different formulations were determined by flow cytometry. Ouabain and digoxin, two cardiotonic steroids, induced ROS production in LLC-PK1 cells, and this induction was inhibited by CoPP pretreatment..

**Ponceau S DNP derivatives**


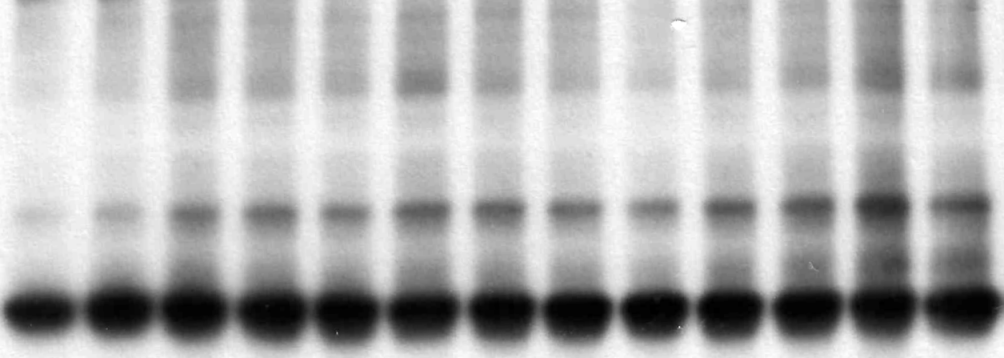

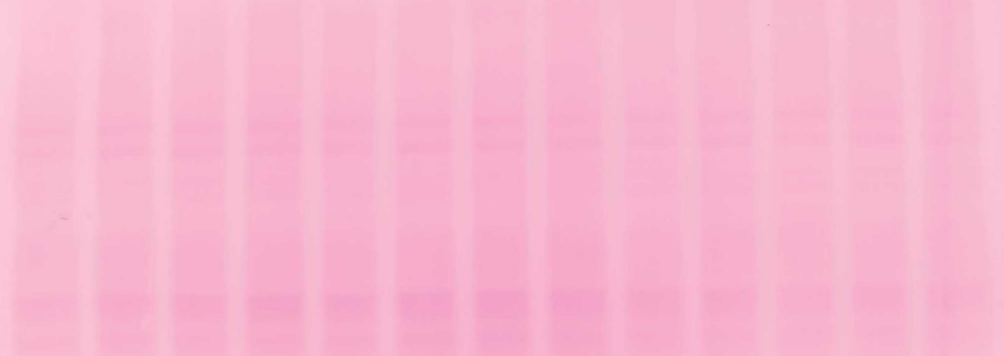


**CTR**

**DMSO**

**Dig,1h**

**CoPP**

**+Dig,1h**

**Dig,2h**

**CoPP**

**+Dig,2h**

**CoPP**

**Figure S9.** In vitro study using HK-2 cells demonstrated that CoPP pretreatment (2uM,24h) inhibited protein carbonylation induced by Digoxin.The Ponceau S stained membrane was used for loading control.(Data represent mean ± SEM; the one-way ANOVA with Tukey’s post-hoc comparison was used for statistical significance. n=3. ^****^*P*<0.001 vs control group; ^#^*P*<0.05 vs Digoxin treated groups, respectively)


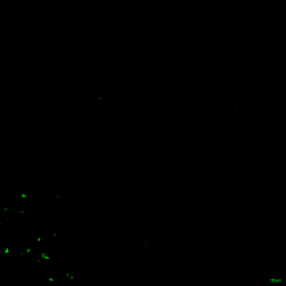

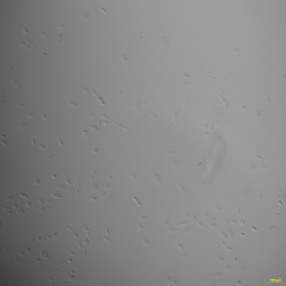

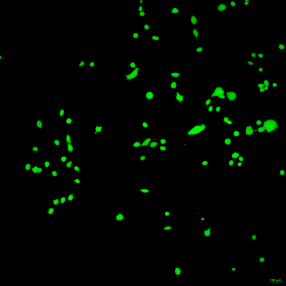

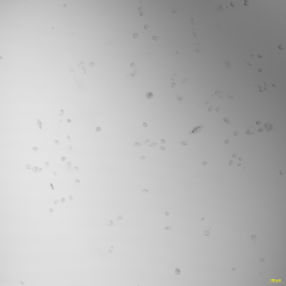

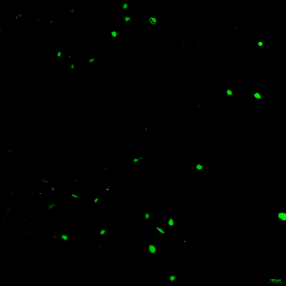

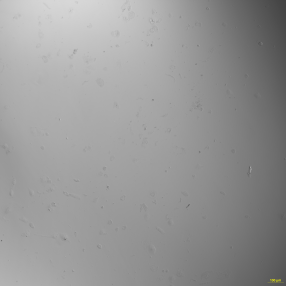

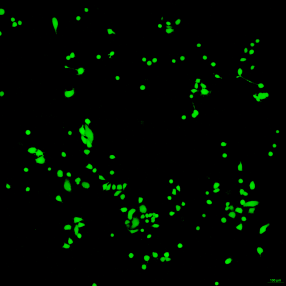

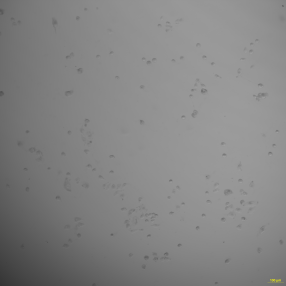


**CTR Ouab Ouab+CoPP CoPP**

**DCFH-DA Phase contrast**

**400μm**

**Figure S10**. Representative ROS fluorescence images of HK-2 cells. Ouabain induced intracellular ROS generation, and this effect was inhibited by pretreatment with CoPP.

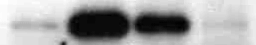


**p-STAT3**


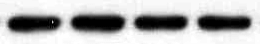


**STAT3**


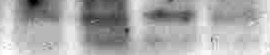


**p-ERK1/2**

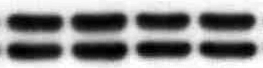


**t-ERK1/2**


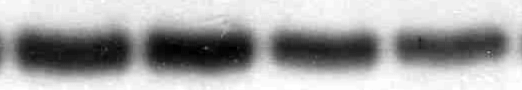


**β-Actin**

**IL-6 (10ng/mL) - + + -**

**CoPP(2μM) - - + +**

**Figure S11**. IL-6 antigen-induced activation of STAT3 and ERK1/2 in LLC-PK1 cells was inhibited by CoPP pretreatment. ERK1/2 activation was expressed as phospho-ERK1/2 / total ERK1/2 (p-ERK1/2 / t-ERK1/2) ratio. STAT3 activation was expressed as phospho-STAT3 / total STAT3 (p-STAT3 / STAT3) ratio.(Data represent mean ± SEM; the one-way ANOVA with Tukey’s post-hoc comparison was used for statistical significance. n=3. ^****^*P*<0.001, ^***^*P*<0.001, ^**^*P*<0.01, ^*^*P*<0.05, respectively.)


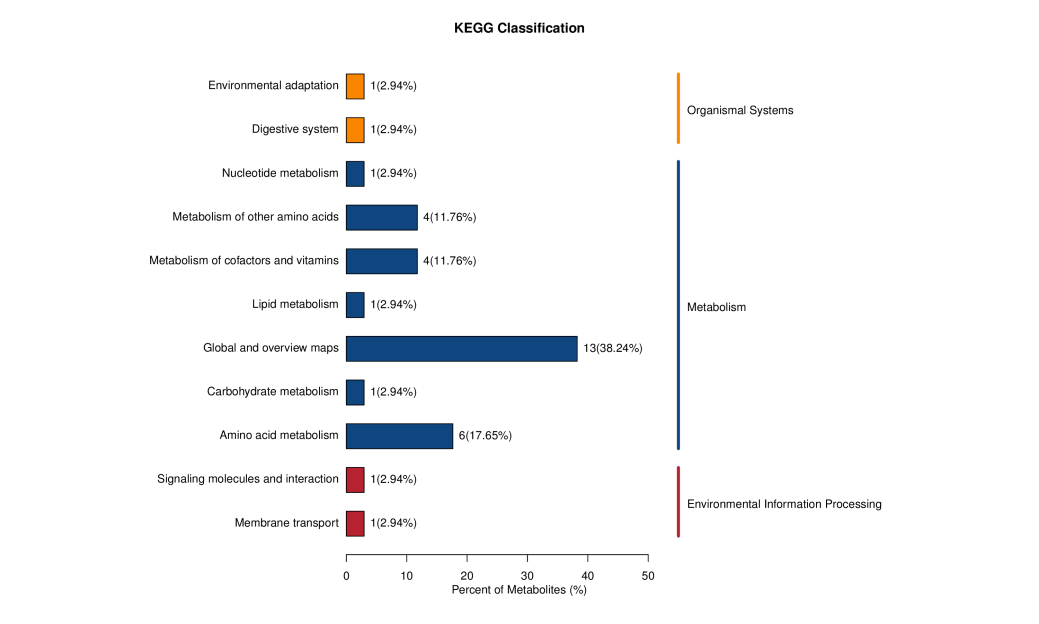


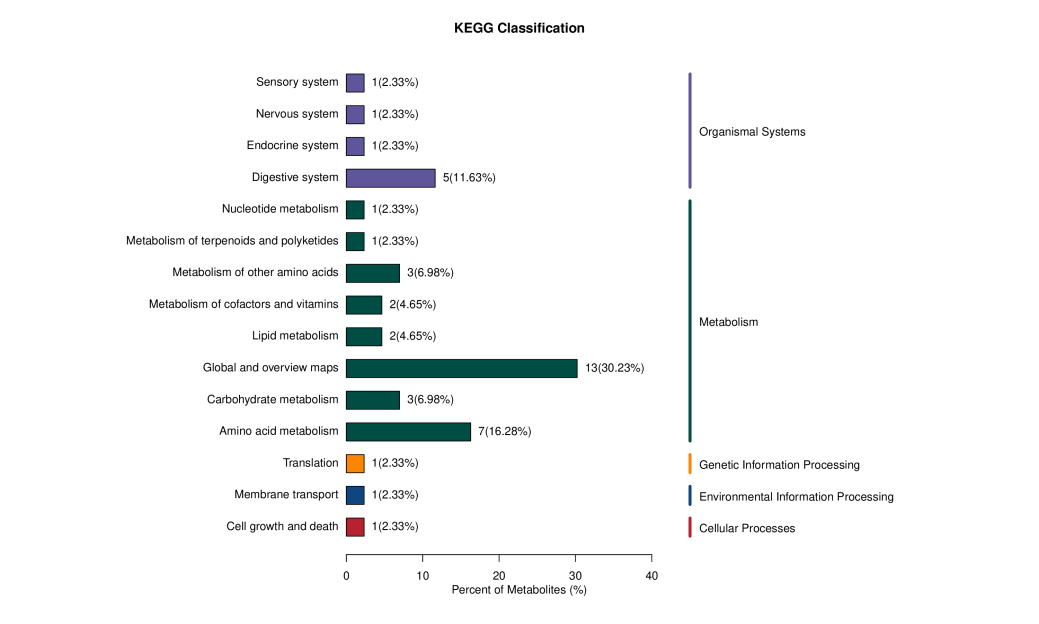
**Figure S12**. KEGG classification diagram of differential metabolites in urine samples in POS ion mode.

**Figure S13**. KEGG classification diagram of differential metabolites in urine samples in NEG ion mode.

|  | LF  (n=6) | LF+HS  (n=6) | LF+HS+CoPP  (n=6) | HF  (n=6) | HF+HS  (n=6) | HF+HS+CoPP  (n=8) |
| --- | --- | --- | --- | --- | --- | --- |
| BW(g) | 27.25±1.81 | 29.67±1.57 | 29.25±0.65 | 43.58±3.34^****^ | 42.67±4.72 | 45.50±2.82 |
| Length (cm) | 10.00±0.00 | 10.00±0.63 | 9.63±0.75 | 10.50±0.89 | 10.08±0.38 | 10.25±0.27 |
| TL (mm) | 18.10±0.49 | 18.27±0.29 | 18.15±0.15 | 18.21±0.12 | 18.34±0.26 | 18.18±0.40 |
| Lee’s index | 3.01±0.07 | 3.10±0.15 | 3.22±0.26 | 3.37±0.28^*^ | 3.46±0.13 | 3.48±0.07 |
| AFI | 33.19±8.54 | 28.50±9.91 | 28.86±5.78 | 89.94±11.22^****^ | 64.11±9.70^$$^ | 89.07±8.67^^^^^ |
| FW /TL | 55.41±17.19 | 46.28±15.99 | 46.54±9.45 | 214.76±27.56^****^ | 150.65±35.54^$$^ | 222.80±23.13^^^^^ |
| KW /TL | 15.61±1.72 | 17.15±0.94 | 15.76±1.81 | 19.10±1.32^**^ | 19.21±2.35 | 18.26±1.48 |
| FBG(mmol/L) | 4.38±0.52 | 4.28±0.53 | 3.55±0.21^#^ | 5.92±1.11^*^ | 5.60±0.74 | 5.80±0.53 |

**Table S1**. Body weight(BW), Length, tibial length(TL), Lee’s index, abdominal fat index(AFI), fat weight /tibial length(FW/TL), kidney weight /tibial length(KW/TL), fasting blood glucose(FBG) of C57BL/6J mice in each group. LF, low fat chow (10 kcal%); HF, high fat chow (60 kcal%); HS, high salt chow (8% NaCl). Value=Mean ± SEM; the two-way ANOVA with Tukey’s post-hoc comparison was used for statistical significance. ^****^*P*<0.001, ^**^*P*<0.01, ^*^*P*<0.05 vs LF; ^#^*P*<0.05 vs LF+HS; ^$$^*P*<0.01 vs HF; ^^^^^*P*< 0.001 vs HF+HS, respectively.

|  | LF  (n=6) | LF+HS  (n=6) | LF+HS+CoPP  (n=6) | HF  (n=6) | HF+HS  (n=6) | HF+HS+CoPP  (n=8) |
| --- | --- | --- | --- | --- | --- | --- |
| Plasma BUN (mmol/L) | 15.62±2.39 | 13.26±5.66^***^ | 15.18±3.00^#^ | 14.78±1.18 | 7.25±2.38^$$$$^ | 6.75±0.95 |
| Plasma CRE (μmol/L) | 69.54±19.80 | 47.93±7.07 | 50.51±13.99 | 61.28±22.10 | 62.52±9.98 | 45.27±11.12 |
| Urine BUN (mmol/L) | 2.84±0.22 | 0.43±0.11^****^ | 0.47±0.06 | 3.71±0.17^****^ | 0.79±0.39^$$$$^ | 0.49±0.26 |
| Urine CRE (μmol/mL) | 5.72±0.17 | 0.77±0.21^****^ | 1.42±0.72 | 5.50±0.08 | 4.40±3.12^$$$$^ | 3.02±1.57 |
| Urine BUN (mmol/24h) | 1.84±0.14 | 2.64±0.38^****^ | 1.89±0.98 | 2.52±0.12^****^ | 1.07±0.74^$$$$^ | 0.93±0.24 |
| Urine CRE (μmol/24h) | 3.72±0.11 | 4.85±1.13 | 4.92±2.04 | 3.74±0.06 | 5.73±0.28^$$$$^ | 5.79±0.69 |
| Ccr  (μl/min) | 22.76±0.68 | 66.67±16.52^*^ | 42.10±7.02 | 28.83±0.45^****^ | 50.97±10.02 | 74.09±11.07^^^ |
| Plasma C_Na+_ (mmol/L) | 157.32±12.11 | 161.04±3.38 | 166.14±7.21 | 157.89±3.97 | 165.46±2.82 | 162.46±3.10 |
| Plasma C_K+_ (μmol/L) | 18.51±1.99 | 16.68±3.97 | 18.96±2.32 | 18.65±2.80 | 17.13±1.81 | 19.38±2.85 |
| Urine C_Na+_ (mmol/mL) | 0.198±0.054 | 0.314±0.053^***^ | 0.150±0.088^##^ | 0.129±0.051 | 0.073±0.025 | 0.054±0.031 |
| Urine C_K+_ (mmol/mL) | 0.411±0.125 | 0.041±0.009^****^ | 0.038±0.011 | 0.209±0.033 | 0.040±0.014^$$$$^ | 0.036±0.016 |
| Urine N_Na+_ (mmol/24h) | 0.221±0.089 | 2.004±0.433^****^ | 0.717±0.665^##^ | 0.088±0.035 | 0.086±0.049 | 0.103±0.037 |
| Urine N_K+_ (mmol/24h) | 0.451±0.181 | 0.258±0.059^*^ | 0.143±0.069^#^ | 0.142±0.022^*^ | 0.051±0.033^$^ | 0.072±0.020 |

**Table S2.** BUN, CRE, Na^+^ and K^+^ concentration in urine or plasma, Ccr, and urinary Na^+^ and K^+^ excretion of C57BL/6J mice in each group.Value=Mean ± SEM; the two-way ANOVA with Tukey’s post-hoc comparison was used for statistical significance. ^****^*P*<0.001, ^***^*P*<0.001, ^*^*P*<0.05 vs LF; ^###^*P*<0.001, ^##^*P*<0.01, ^#^*P*<0.05 vs LF+HS; ^$$$$^*P*<0.0001, ^$^*P*<0.05 vs HF; ^^^*P* < 0.05 vs HF+HS, respectively.

**Table S3**. Top ten differential metabolites in urine samples of HF+HS and HF+HS+CoPP in POS and NEG ion modes obtained by volcano plots.

**Table S4**. Differential metabolites in urine samples of mice were associated with 36 KEGG pathways.

**Table S5**. The 7 metabolic pathways associated with oxidative stress enriched by KEGG involved 15 metabolites.

**Table S6**. Correlation coefficients of differential metabolites with indices of obesity, oxidative stress and sodium metabolism.
